# Combined exposure to non-antibiotic pharmaceutics and antibiotics in the gut synergistically promote the development of multi-drug-resistance in *Escherichia coli*

**DOI:** 10.1080/19490976.2021.2018901

**Published:** 2022-01-11

**Authors:** Danyang Shi, Han Hao, Zilin Wei, Dong Yang, Jing Yin, Haibei Li, Zhengshan Chen, Zhongwei Yang, Tianjiao Chen, Shuqing Zhou, Haiyan Wu, Junwen Li, Min Jin

**Affiliations:** Key Laboratory of Risk Assessment and Control for Environment & Food Safety, Tianjin Institute of Environmental & Operational Medicine, Tianjin, China

**Keywords:** Non-antibiotic pharmaceutics, antibiotics, synergism, antibiotic-resistant bacteria

## Abstract

The gut microbiota represents an important reservoir of antibiotic-resistant bacteria (ARB), which poses a significant threat to public health. However, little is known about the emergence of ARB in the gut after the combined exposure to antibiotics and non-antibiotic pharmaceutics. Here, *Escherichia coli*, a common opportunistic pathogen in the gut microbiota, was exposed to the antidepressant duloxetine (2.5 µg/L–25 mg/L) and/or chloramphenicol (6 µg/L–4 mg/L). The resistant strains were isolated to determine the minimum inhibition concentration (MIC) of 29 antibiotics. Then, genome-wide DNA sequencing, global transcriptomic sequencing, and real-time quantitative polymerase chain reaction were performed to quantify the synergy between duloxetine and chloramphenicol. Combined exposure synergistically increased the mutation frequency of chloramphenicol resistance by 2.45–9.01 fold compared with the independent exposure. A combination index reaching 187.7 indicated strong duloxetine and chloramphenicol synergy. The resultant mutants presented heritable enhanced resistance to 12 antibiotics and became ARB to eight antibiotics. Furthermore, combined exposure significantly increased the transcriptomic expression of *acrA, acrB*, and *marA* in *E. coli*, and generated a more robust oxidative stress response. Together with the occurrence of DNA mutations in *marR* in the mutants, stronger triggers to the AcrAB-TolC transport system and the MlaFEDB ABC transporter via reactive oxygen species (ROS)-induced mutagenesis, verified by gene knockout, contributed to the synergistic enhancement of antibiotic resistance in the combined exposure group. Regardless of whether their formation was induced by duloxetine, chloramphenicol, or their combination, the *E. coli* mutants showed 1.1–1.7-fold increases in the expression levels of *acrA, acrB, acrZ, mdtE*, and *mdtF*. This pattern indicated that the mutants shared the same resistance mechanisms against chloramphenicol, involving the improved efflux pumps AcrAB-TolC and *mdtEF*. Our findings demonstrated that antibiotics and non-antibiotic pharmaceutics synergistically accelerate the evolution of ARB and may enhance their spread.

## Introduction

The appearance of antibiotic-resistant bacteria (ARB) has resulted in the failure to treat serious infectious diseases in clinics. In the EU, 25,000 people die each year from infections caused by ARB.^1^ It is alarming that if adequate measures are not taken to control the spread of ARB, an additional 10 million people may be killed globally each year.^2^ Consequently, ARB have become a severe worldwide threat to public health.

The gut microbiota is an important reservoir of ARB; this could be related to the overuse or misuse of antibiotics in hospitals and the contamination of food and drinking water antibiotics.^3–6^ A clinical trial found that antibiotic concentrations in the guts of healthy individuals reached 26.51 ± 33.48 ng/g, which could put severe antibiotic pressure on gut microbiota and result in a global increase in the dissemination of ARB.^7,8^ Above all, with the discovery of the strengthened resistance of *Escherichia coli* to multiple antibiotics, e.g., tetracycline, chloramphenicol, cefalexin, levofloxacin, and norfloxacin, after exposure to the antidepressant fluoxetine,^9^ the contribution of non-antibiotic pharmaceuticals, particularly antidepressant drugs, toward the dissemination of ARB has become an urgent concern due to their widespread use.

Non-antibiotic pharmaceuticals typically have a prolonged usage. As a result, patients could consume non-antibiotic pharmaceuticals and antibiotics together, resulting in the gut microbiota being exposed to both. The combined persistence of these drugs may pose a cumulative or magnified pressure on microorganisms. Therefore, the significant risk of ARB dissemination posed by the coexistence of non-antibiotic pharmaceuticals and antibiotics makes it imperative that we better understand the emergence of ARB under the combined exposure to non-antibiotic pharmaceuticals and antibiotics. Previous studies have reported a synergistic effect between the antidepressant sertraline with the antibiotic tetracycline against tetracycline-resistant *E. coli*,^10^ as well as the synergistic interactions between the antibiotic polymyxin B and non-antibiotics, which provide alternative strategies for difficult-to-treat infections.^11^ However, whether the combined exposure synergistically promotes the evolution of ARB remains unclear.

Depression has become one of the most prevalent disorders worldwide, impacting >16% of the world’s population and leading to the massive use of antidepressant drugs.^12–14^ In this study, duloxetine, the leading antidepressant pharmaceutical, and chloramphenicol were selected as a representative non-antibiotic pharmaceutical and antibiotic, respectively, to investigate their combined effects on antibiotic resistance in *E. coli*, an opportunistic pathogen in the gut microbiota. The mechanisms underlying their collaborative enhancement of resistance to antibiotics were revealed by genomic sequencing, together with transcriptomic analysis, and were further verified by real-time quantitative polymerase chain reaction (RT-qPCR) and clustered regularly interspaced palindromic repeat and Cas9 (CRISPR/Cas9) gene editing.^15^ To the best of our knowledge, this work, for the first time, shows the synergistic induction of the resistance to multiple antibiotics by non-antibiotic pharmaceuticals and antibiotics, specifically at residual levels. Our findings will broaden our understanding of the emergence of antibiotic resistance and provide a novel theoretical basis for the dissemination of ARB.

## Results

### Combined exposure to duloxetine and chloramphenicol can synergistically induce multiple antibiotic resistance in *E. coli*

To reveal the effects of duloxetine and/or chloramphenicol exposure on antibiotic resistance, *E. coli* was exposed to duloxetine at concentrations ranging from 2.5 µg/L to 25 mg/L and/or chloramphenicol at a sublethal concentration (6 µg/L–4 mg/L).

**Figure 1.**
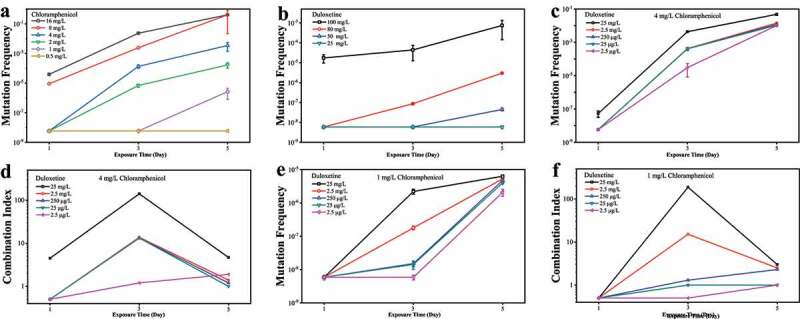
**The chloramphenicol-resistant mutation in *E. coli* exposed to chloramphenicol or/and duloxetine**. The baseline conditions were as follows: 30 µL of fresh overnight cultured wild-type *E. coli* K12 was inoculated into 2.97 mL LB medium containing chloramphenicol (A, 0.5–16 mg/L) or duloxetine (B, 25–100 mg/L) or combined (C, 4 mg/L chloramphenicol and 2.5 µg/L–25 mg/L duloxetine) or combined (E, 1 mg/L chloramphenicol and 2.5 µg/L–25 mg/L duloxetine). All experiments were performed in triplicate. The combination indexes between chloramphenicol and duloxetine were calculated when *E. coli* K12 experienced combined exposure to 4 mg/L chloramphenicol (d) or 1 mg/L chloramphenicol (f) with 2.5 µg/L–25 mg/L duloxetine.

The results showed that chloramphenicol resistance occurred in *E. coli* exposed solely to chloramphenicol (Figure 1(a)). Furthermore, they presented a dose-response pattern, and the mutation frequency declined sharply with the decrease in chloramphenicol exposure levels from 16 to 0.5 mg/L (*p* < .01). At the exposure level of 16 mg/L chloramphenicol for five days, the mutation frequencies of chloramphenicol resistance increased by at least 7.0 × 10^7^-fold to 0.41 (Table S1), compared with the spontaneous mutation frequency of chloramphenicol, which was less than 5.9 × 10^−9^ (no spontaneous chloramphenicol-resistant mutants were observed on the plates containing 16 mg/L chloramphenicol). However, when the chloramphenicol dose was lowered to 1 mg/L, the mutation frequencies dramatically decreased to 2.6 × 10^−6^ over the same period. Furthermore, the bacterial mutation frequency of chloramphenicol also showed a time-dependent pattern. While no increase in the mutation frequency was found on day 1 in case of 4 mg/L chloramphenicol, the mutation frequency of chloramphenicol increased to 1.3 × 10^–4^ on day 3 (clones named C_4_-3d) and to 3.4 × 10^−3^ on day 5. Evidently, the higher the exposure concentration, the faster was the increase in the mutation frequency over time. The resistant *E. coli* began to grow on chloramphenicol selecting-plates on day 1, day 3, and day 5 for for 8 mg/L, 4 mg/L, and 1 mg/L chloramphenicol exposure, respectively. No significant increase in the mutation frequency occurred even if *E. coli* was exposed to 6 μg/L chloramphenicol for 50 days.

For the first time, the antidepressant duloxetine was also found to induce chloramphenicol resistance in *E. coli*, showing dose- and time-dependent patterns similar to those observed with chloramphenicol exposure (Figure 1(b), Table S2). After one-day exposure to 100 mg/L duloxetine, *E. coli* obtained resistance to chloramphenicol with a mutation frequency increase of at least 3.0 × 10^3^-fold to 1.7 × 10^−5^ (clones named D_100_-1d), while it reached at least a 1.3 × 10^5^-fold increase to 7.4 × 10^−4^ over a five-day exposure period. However, when the concentrations of duloxetine decreased to 50 mg/L, significant decreases in mutation frequency could be found compared with those exposed to 100 mg/L for the same duration (*p* < .05). Overall, the resistant *E. coli* started growing on chloramphenicol plates on day 1 in the presence of 100 mg/L duloxetine and day 3 in the presence of 80 mg/L duloxetine. However, when the duloxetine concentration was 2.5 μg/L, no colonies were found on chloramphenicol plates even if the exposure time was prolonged to 50 days.

Considering that both the concentrations of antidepressants and antibiotics in the gut environment were lowered to the level of μg/L to mg/L,^7,16^ the effects of combined exposure to duloxetine and chloramphenicol at gut environmental concentration on the mutation frequency of chloramphenicol-resistance was further tested. Surprisingly, after 30 days of combined exposure to 25 μg/L duloxetine and 60 μg/L chloramphenicol and 50 days of combined exposure to 2.5 μg/L duloxetine and 6 μg/L chloramphenicol, resistant *E. coli* (clones named D_0.025_-C_0.06_–30d and D_0.0025_-C_0.006_–50d) started growing on chloramphenicol-resistance plates and the frequency of chloramphenicol resistance mutations increased by an average of at least 2.5-fold to 1.4 × 10^−8^, indicating that the combined exposure to duloxetine and chloramphenicol can synergistically promote bacterial resistance to chloramphenicol, compared with the exposure to duloxetine or chloramphenicol independently. To determine the combination index between duloxetine and chloramphenicol with regard to the mutation frequency of chloramphenicol, the effects of combined exposures to 1 or 4 mg/L chloramphenicol and duloxetine in the range of 0.025–25 mg/L were observed. Figure 1(c) shows that the combined exposure to 25 mg/L duloxetine and 4 mg/L chloramphenicol for one day increased the mutation frequency of chloramphenicol by at least 9.1-fold to 5.3 × 10^−8^ (clones named D_25_-C_4_-1d, Table S3), while no significant difference occurred between the independent exposure to 25 mg/L duloxetine or 4 mg/L chloramphenicol with the spontaneous mutation frequency of chloramphenicol. The combination index of duloxetine and chloramphenicol was 4.5 (Figure 1(d)). Figure 1(e) shows that the mutation frequency increased by at least 375-fold to 2.2 × 10^−6^ on day 3 for *E. coli* treated with combined exposure to 25 mg/L duloxetine and 1 mg/L chloramphenicol, and the combination index reached 187.7 (Figure 1(f)), which clearly shows that duloxetine and chloramphenicol could synergistically induce resistance to multiple antibiotics in *E. coli*.

**Figure 2.**
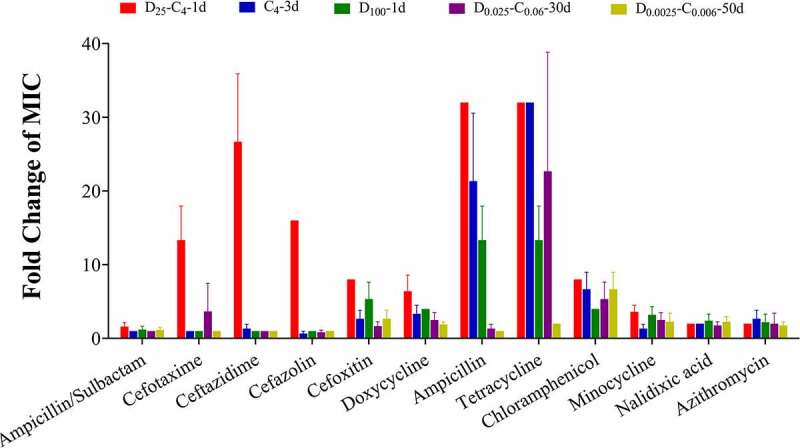
**Fold differences of the MICs against representative antibiotics for *E. coli* mutants selected from combined or independent exposure to duloxetine and chloramphenicol (n = 3)**. The MICs of wild-type *E. coli* K12 and 5–8 clones of resistant mutants D_25_-C_4_-1d, D_100_-1d, C_4_-3d, D_0.025_-C_0.06_–30d, and D_0.0025_-C_0.006_–50d against 29 kinds of antibiotics were determined using the Thermo Scientific^TM^ Sensititre^TM^ Susceptibility Testing System (Thermo Fisher Scientific, USA). The fold differences of the MICs against antibiotics between mutants and wild-type *E. coli* were calculated.

The MICs of 29 types of representative antibiotics were tested for 5–8 isolates obtained from the above chloramphenicol plates that were treated with independent or combined exposure (Tables S4, S5–1 and S5-2). The result shows that the MIC of 12 antibiotics for the clone D_25_-C_4_-1d significantly increased by up to 32-fold, compared with that of wild-type *E. coli* (*p* < .05). Above all, the MICs of 6 antibiotics (ampicillin, cefotaxime, ceftazidime, cefazolin, cefoxitin, and doxycycline) were significantly higher for the clones D_25_-C_4_-1d than for the clones C_4_-3d and D_100_-1d (*p* < .05). The clones D_25_-C_4_-1d developed multiple antibiotic resistance to tetracycline, cefazolin, cefoxitin, ampicillin, and chloramphenicol. The clones C_4_-3d developed resistance to tetracycline, ampicillin, and chloramphenicol. The clones D_100_-1d developed resistance to cefoxitin and chloramphenicol. However, the clones D_0.025_-C_0.06_–30d and D_0.0025_-C_0.006_–50d only gained significantly higher MICs of 6 antibiotics (tetracycline, cefoxitin, doxycycline, chloramphenicol, minocycline and nalidixic acid) compared with the wild-type *E. coli* K12 (*p* < .05). The clones D_0.025_-C_0.06_–30d developed resistance to tetracycline and chloramphenicol. The clones D_0.0025_-C_0.006_–50d only developed resistance to chloramphenicol (Figure 2). The MICs of antibiotics for all the isolates remained unchanged even after 10 passages through the lysogeny broth (LB) medium (Table S4), suggesting that antibiotic-resistant isolates displayed the genetic stability of antibiotic resistance, which would result in the vertical transfer of antibiotic-resistant genes (ARGs).

### The combined exposure to duloxetine and chloramphenicol synergistically affected several pathways analyzed by global transcriptomic sequencing

**Figure 3.**
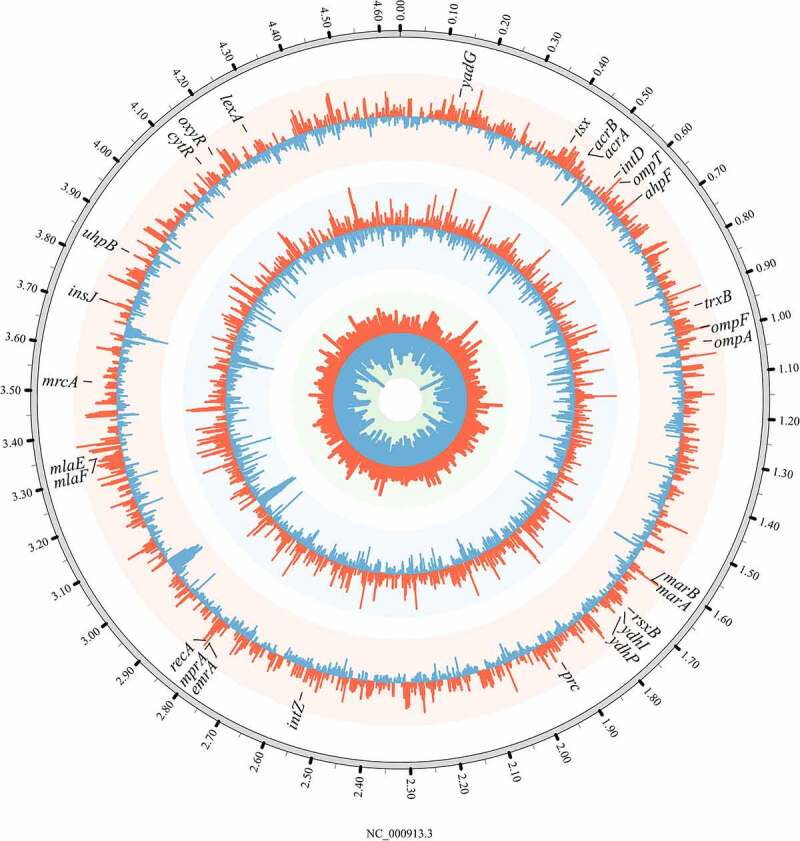
**Transcriptional response relating to the antibiotic resistance in wild-type*E. coli* K12 that treated with 25 mg/L duloxetine or/and 4 mg/L chloramphenicol exposure (n = 3)**. A circular representation of the transcriptional profile. The outermost circle represents the *E. coli* K12 genome. The circles from the inner outward correspond to the expression of each gene in wild-type *E. coli* under exposure to 25 mg/L fluoxetine, 4 mg/L chloramphenicol, and their combination, respectively. The red lines in each circle represent the up-regulation gene expression (log_2_ > 1), while the blue lines represent the downregulation of mRNA expression (log_2_ < −1). The representative genes are marked at the appropriate genomic position.

Global transcriptomic sequencing analyses were performed to study the mechanism underlying the synergistic enhancement of resistance to chloramphenicol by duloxetine and chloramphenicol in *E. coli* (Figure 3 and S1). For *E. coli* treated with combined exposure to 25 mg/L duloxetine and 4 mg/L chloramphenicol, multiple genes were significantly upregulated by more than 2-fold, including at least 26 genes related to bacterial antibiotic resistance, 23 genes related to DNA repair/replication, and nine genes related to oxidative stress, in comparison to *E. coli* treated independently with 25 mg/L duloxetine or 4 mg/L chloramphenicol. Each category of pathways are analyzed individually as follows to further reveal the mechanisms underlying the synergistic effects of duloxetine and chloramphenicol through their combined exposure.

### The combined exposure to duloxetine and chloramphenicol significantly promoted the overexpression of the bacterial efflux pumps AcrAB-TolC and MlaFEDB

According to the transcriptomic analysis, the genes related to bacterial antibiotic resistance that were upregulated after the combined exposure include the efflux pump system/transporter (e.g., *acrA* and *acrB*) and their regulatory genes (e.g., *marR* and *marA*), membrane pore protein genes (e.g., *ompA* and *ompF*), mobile element-associated genes (e.g., *intD, intZ*, and *intJ*), penicillin-resistance genes (*prc* and *mrcA*) etc. (Table S6, Figure. S1). Notably, none of the genes showed significant changes in expression after the independent exposure to8 25 mg/L duloxetine, but 13 genes showed notably upregulated expression after the independent exposure to 4 mg/L chloramphenicol

Among the efflux pump system and its regulatory genes, the expression levels of *acrA* and *acrB* increased by around 4-fold after the combined exposure to 25 mg/L duloxetine and 4 mg/L chloramphenicol. The periplasmic fusion protein AcrA and drug proton transporter AcrB, together with the outer membrane channel protein TolC, forms the active external pump AcrAB-TolC transport system of *E. coli*. AcrAB-TolC belongs to the resistance-nodulation-cell division (RND) superfamily of the *E. coli* efflux pump system, which helps bacteria resist various antibiotics, such as fluoroquinolones, tetracyclines, and cephalosporins.^17–20^ Above all, the expression of the gene *marA*, which modulates the transcription of the *acrAB* operon, increased by 2.8-fold in the combined exposure group. In contrast, in the independent exposure group, the expressions levels of *marA* were not significantly increased. The genes *mlaD, mlaE*, and *mlaF* belonging to the MlaFEDB ABC transporter, which drives phospholipid trafficking across the bacterial envelope to maintain the outer membrane integrity,^21,22^were also found to be significantly upregulated by 3.7–4.6-fold in the combined exposure group, according to the global transcriptomic sequencing analyses.

**Figure 4.**
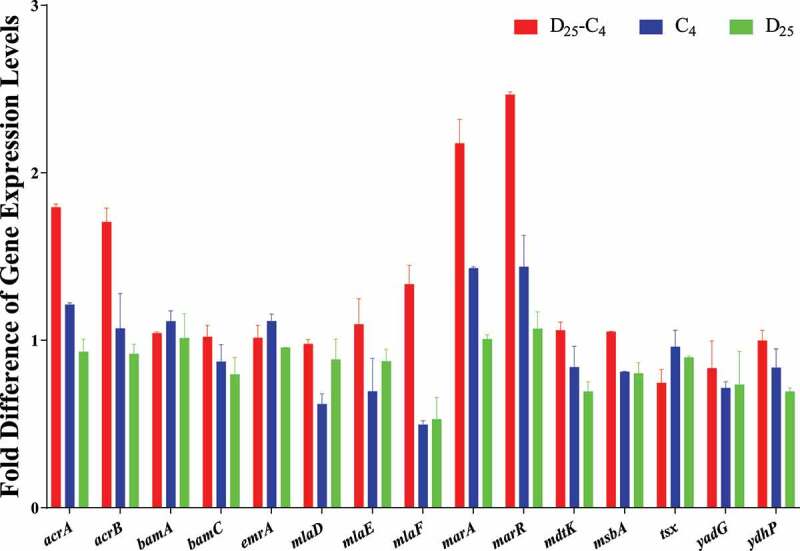
**Quantification of the differentially expressed genes in wild-type*E. coli* K12 treated with 25 mg/L duloxetine or/and 4 mg/L chloramphenicol exposure (n = 3)**. The baseline conditions were: 100 µL of fresh overnight cultured wild-type *E. coli* K12 was inoculated into 10 mL LB medium containing 25 mg/L duloxetine or/and 4 mg/L chloramphenicol and cultured for 10 h at 37°C. Then, the relative concentration of interesting genes was assayed by RT- qPCR. As controls, bacteria without any exposure were also assayed and the fold difference of the expression levels of interesting genes between them were calculated.

To verify the contribution of the efflux pump system to the synergy between duloxetine and chloramphenicol, the expression of key genes in the transcriptome (*acrA, acrB, bamA, bamC, emrA, mlaD, mlaE, mlaF, marA, marR, mdtK, msbA, tsx, yadG*, and *ydhP*) were tested using qPCR. The results demonstrated that the expression levels of *acrA, acrB*, and *marA* in *E. coli* treated with the combined exposure to 25 mg/L duloxetine and 4 mg/L chloramphenicol were upregulated 1.7–2.2-fold; these were significantly higher than those observed after the exposure to both agents independently (*p* < .05, Figure 4, Table S7). Meanwhile, after validation with qPCR, the expression level of *mlaF* in the combined exposure group was found to be 1.3-fold higher (*p* < .05, Table S8); this was significantly higher than that observed after the exposure to both agents independently. Therefore, the enhanced expression of the AcrAB-TolC transport system- and MlaFEDB-associated genes in the combined exposure group may contribute to the synergistic promotion of antibiotic resistance in *E. coli*.

To further validate the role of the AcrAB-TolC and MlaFEDB systems in the synergy between duloxetine and chloramphenicol, the genes belonging to these systems (*marA, acrA, acrB, mlaD, malE*, and *mlaF*) were knocked out in wild-type *E. coli* K12 using the CRISPR/Cas system, followed by the exposure to duloxetine and/or chloramphenicol (Table S8). The knockout of the *marA* gene rendered *E. coli* completely devoid of drug resistance mutations after exposure to duloxetine and/or chloramphenicol. However, *E. coli* still mutates under drug exposure after knocking out the genes *acrA, acrB, mlaD, mlaE*, or *mlaF*, suggesting that the AcrAB-TolC transport system and the MlaFEDB ABC transporter act together in the synergistic promotion of antibiotic resistance in *E. coli*.

### Combined exposure resulted in a stronger oxidative stress response in *E. coli* compared with the independent exposure

The expression of antioxidant genes in response to oxidative stress was also analyzed by transcriptomic sequencing (Table S9, Figure. S1). For *E. coli*, the *oxyR* regulon (primarily associated with response to hydrogen peroxide (H_2_O_2_)) and *soxRS* regulon (primarily associated with response to superoxide (O_2_^−^)) defends cells against the damage caused by reactive oxygen species (ROS).^23,24^ The results show that not only did the expression of *oxyR* increase by 2.1-fold in the combined exposure group, but the expression levels of the downstream genes upregulated by the OxyR redox response system were also elevated: *grxA* (Glutaredoxin A; by 27.9-fold), *ahpF* (alkyl hydroperoxide reductase; by 8.6-fold), *fur* (ferric iron uptake regulon transcriptional repressor; by 2.3-fold), and *trxB* (thioredoxin reductase; by 2.8-fold). Overexpression of these genes conferred the abilities of peroxide metabolism, redox balance, peroxide protection, glutathione synthesis and reduction, DNA protection under oxidative stress, etc., onto the strains.^25^ Therefore, the OxyR redox response system may play an important protective role in *E. coli* exposed to duloxetine and chloramphenicol. Additionally, the expression levels of the components of the rsxABCDGE gene cluster, which encodes a SoxR-reducing system to turn off the SoxR-mediated induction of the activation of the transcription factor SoxS in the absence of oxidizing agents,^26^ were elevated by 2.3–4.3-fold, indicating that *soxS* transcription may not be constitutively activated here. Taken together, there was no significant increase in the expression levels of the aforementioned genes after the independent exposure to 25 mg/L duloxetine, and only the expression level of *ahpF* increased by 4-fold after the independent exposure to 4 mg/L chloramphenicol; correspondingly, combined exposure resulted in a more robust oxidative stress response in *E. coli* than the independent exposure.

**Figure 5.**
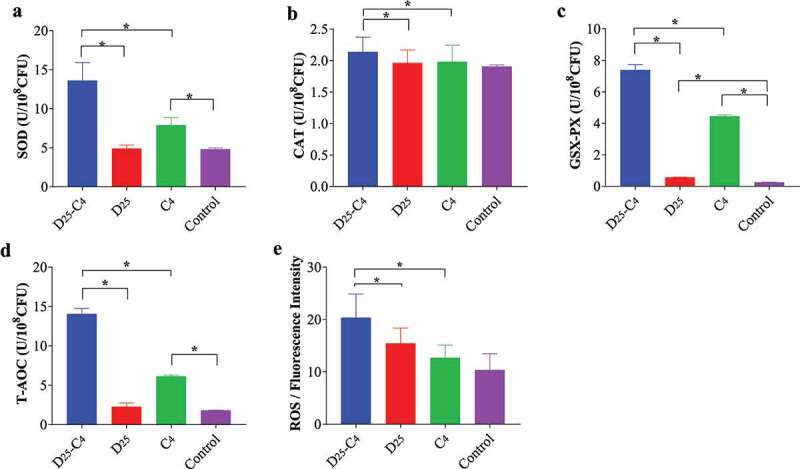
**Oxidative stress response in wild-type*E. coli* K12 treated with 25 mg/L duloxetine or/and 4 mg/L chloramphenicol exposure (n = 3)**. The baseline conditions were: Bacteria suspension cultured in an LB medium containing 25 mg/L duloxetine or/and 4 mg/L chloramphenicol (D_25_-C_4_, D_25_, C_4_) for 10 h at 37°C was centrifuged at 10,000 × *g*, 4°C for 1 min and then the collected pellets were suspended in 0.2 mol/L phosphate-buffered saline (PBS) and sonicated for three minutes under an ice-water bath. After centrifugation at 5,000 × *g*, 4°C for 3 min, the contents of superoxide dismutase (SOD), catalase (CAT), glutathione peroxidase (GSH-PX), and total antioxidant capacity (T-AOC) in the supernatant were assayed (a-d). The reactive oxygen species (ROS) was measured (e) using the DCF-DA/H_2_DCFDA-cellular ROS detection assay kit (Abcam, USA). As controls, bacteria without any exposure were also assayed.

To validate the oxidative stress response in *E. coli* treated with 25 mg/L duloxetine and/or 4 mg/L chloramphenicol, the ROS production and activities of the cellular antioxidant systems were further analyzed (Figure 5, Table S10). After the combined exposure to 25 mg/L duloxetine and 4 mg/L chloramphenicol, the ROS content increased by 2-fold, compared with that in the control group (*p* < .05). In comparison, after 25 mg/L duloxetine or 4 mg/L chloramphenicol treatment, there was no significant difference in the ROS content (*p* > .05). Meanwhile, to protect the bacteria from ROS-induced destruction, cellular antioxidant systems, including superoxide dismutase (SOD), catalase (CAT), glutathione peroxidase (GSH-PX), and total antioxidant capacity (T-AOC) were activated, correspondingly. After the combined exposure to 25 mg/L duloxetine and 4 mg/L chloramphenicol, the GSH-PX activity increased by 29.6-fold, the SOD activity, by 2.8-fold, and the T-AOC, by 7.7-fold, compared with those in the control group (*p* < .05, Table S10). Above all, ROS production, SOD activity, GSX-PH activity, CAT activity, and T-AOC of the combined exposure group were significantly higher than those of the two independent-exposure groups (*p* < .05).

### Combined exposure resulted in differential DNA mutations in *E. coli* compared with independent exposure

After analyzing the transcriptomic expression of genes involved in DNA repair in response to the combined exposure to duloxetine and chloramphenicol by transcriptomic sequencing (Figure 3 and S1 and Table S11), the exposed *E. coli* were revealed to show a dramatic increase in the *lexA* and *recA* expression levels by about 2.6-fold, indicating that combined exposure damaged *E. coli* DNA and activated its SOS repair system to repair DNA for survival. Meanwhile, the expression levels of genes related to mismatch repair proteins, e.g., *mutL* and *mutS*, and exonucleases, e.g., *recX, recC, recJ, recF, xseA*, and *xthA*, were upregulated by 2.3–2.6-fold and 2.1–2.8-fold, respectively; this may account for the increased mutation rate in the bacteria exposed to both drugs. Otherwise, the expression levels of none of these genes changed significantly in *E. coli* treated with 25 mg/L duloxetine exposure. The genes *rne, obgE, ybfE*, and *ydjM* were significantly upregulated by 2.6–4.6-fold in *E. coli* treated with 4 mg/L chloramphenicol exposure; the expression levels of these genes were lower than those in *E. coli* treated with the combined exposure.

To observe if the mutations occurred during the exposure, genome sequencing targeting the resistant isolates was performed. No chloramphenicol-resistant clones occurred in case of *E. coli* exposed to 25 mg/L duloxetine or 4 mg/L chloramphenicol for one day, 25 µg/L duloxetine or 60 µg/L chloramphenicol for 30 days, or 2.5 µg/L duloxetine or 6 µg/L chloramphenicol for 50 days; therefore, we sequenced the genome of the resistant isolates induced by their combined exposure. The findings revealed that all the isolates presented a mutation type in *marR*, which is a repressor of the *marORAB* operon (Table 1). This includes base insertion or deletion leading to an early stop codon in *marR* or a substitution of a single nucleotide polymorphism in *marR*. Otherwise, no mutation occurred in the genes associated with DNA replication/repair or oxidative stress systems. *E. coli* K12 with *marA* knock out showed no occurrence of mutations on the chloramphenicol-containing plates after the combined exposure to 25 mg/L duloxetine and 4 mg/L chloramphenicol; this suggests that in this study, the mutation in *marR* enabled the development of antibiotic resistance.

**Table 1.** Gene *marR* mutations in chloramphenicol-resistant *E. coli* isolations induced by combined exposure

Isolations	Position	Types	Codon_mutate	AA_mutate
D_25_-C_4_-1d Clone1	1619260	SNP	C→A(TGC→TGA)	C<->U
D_25_-C_4_-1d Clone2	1619389	I7	CGAACCC	
D_25_-C_4_-1d Clone3	1619389	I7	CGAACCC	
D_0.025_-C_0.06_–30d Clone1	1619120	I20	TACTTGCCAGGGCAACTAAT	
D_0.025_-C_0.06_–30d Clone2	1619120	I20	TACTTGCCAGGGCAACTAAT	
D_0.025_-C_0.06_–30d Clone3	1619120	I20	TACTTGCCAGGGCAACTAAT	
D_0.0025_-C_0.006_–50d Clone1	1619430	SNP	G→A(GGC→GAC)	G<->D
D_0.0025_-C_0.006_–50d Clone2	1619379	D1	T	
D_0.0025_-C_0.006_–50d Clone3	1619496	D1	A	

### The mechanism underlying the resistance to chloramphenicol was the same in the *E. coli* mutants

**Figure 6.**
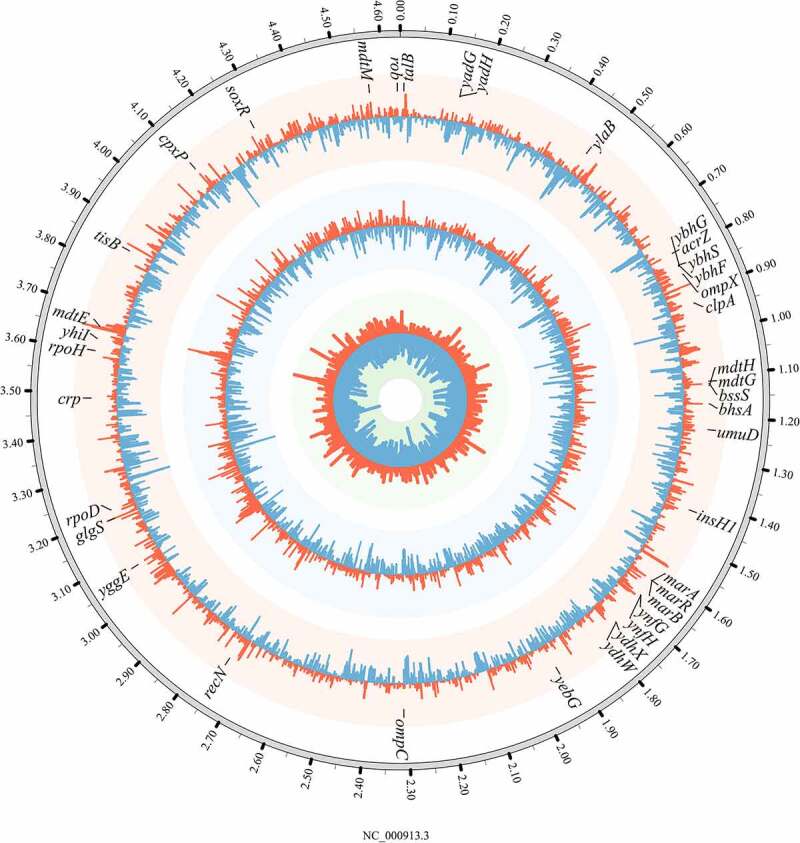
**Transcriptional response to 4 mg/L chloramphenicol exposure in *E. coli* K12 mutants (n = 3)**. A circular representation of the transcriptional profile. The outermost circle represents the full strain *E. coli* K12 genome. The circles from the inner outward correspond to the expression of each gene in *E. coli* mutants D_100_-1d, C_4_-3d, and D_25_-C_4_-1d under exposure to 4 mg/L chloramphenicol, respectively. The red lines in each circle represent the upregulation gene expression (log_2_ > 1), while the blue lines represent the downregulation of mRNA expression (log_2_ < −1). The representative genes are marked at the appropriate genomic position.

To investigate the mechanisms underlying the chloramphenicol resistance of *E. coli* mutants isolated from combined or independent exposure, the *E. coli* mutants (D_100_-1d, C_4_-3d, and D_25_-C_4_-1d) were cultured in the LB broth containing 4 mg/L chloramphenicol for 10 h at 37°C and were then subjected to transcriptomic analyses. According to the transcriptional profile analysis, all mutants presented differential expression of genes associated with the drug efflux system and stress defense when subjected to the 4 mg/L chloramphenicol treatment (Tables S12 and S13 and Figure 6), but no significant differences were found between the expression levels of these genes in the *E. coli* mutants. This observation indicates that the mechanism underlying chloramphenicol resistance was the same for all the mutants, regardless of whether they were induced by duloxetine, chloramphenicol, or their combination.

**Figure 7.**
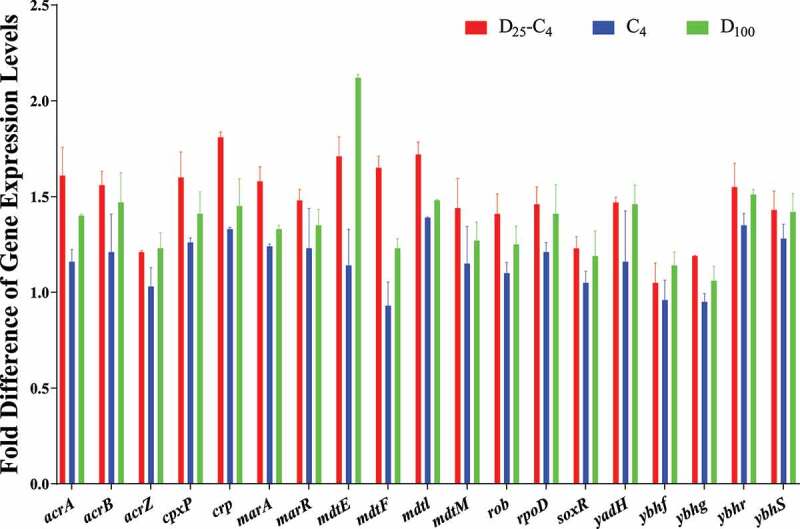
**Differential expression of genes in *E. coli* mutants when exposed to 4 mg/L Chloramphenicol**. 100 µL of fresh overnight cultured wild-type *E. coli* (controls) or *E. coli* mutants D_25_-C_4_-1d, D_100_-1d, and C_4_-3d isolated from the combined exposure (25 mg/L duloxetine and 4 mg/L Chloramphenicol) and sole exposure to either duloxetine (100 mg/L) or chloramphenicol (4 mg/L) groups were inoculated into 10 mL LB containing 4 mg/L chloramphenicol for 10 h at 37°C. Then, the relative concentrations of interesting genes were assayed by RT- qPCR. The fold differences in the expression levels of interesting genes between various mutants and wild-type strains were calculated.

Among the 31 ARGs that may contribute to the enhanced chloramphenicol resistance for mutants induced by the combined exposure, the genes associated with the multi-drug efflux system transporter MdtEF, which confers resistance to compounds such as erythromycin, doxorubicin, ethidium bromide, deoxycholate, and benzalkonium^27,28^ were upregulated (*mdtE* by 8.6-fold and *mdt*F by 2.0-fold). The gene *mdtM*, which encodes a 410-amino acid residue protein that belongs to the large and ubiquitous major facilitator superfamily (MFS),^29^ was upregulated by 12.1-fold. Above all, *rob, marA*, and *crp*, which transcriptionally activate the *acrAB* operon,^30^ were upregulated by 2.1–13.0-fold. The gene *acrZ*, which encodes the small protein AcrZ in *E. coli* to interact with the transmembrane portion of the multi-drug efflux pump AcrB and increases the bacterium’s resistance to a subset of the antibiotic substrates of that transporter,^31,32^ was upregulated by 7.5-fold. Figure 7 further validated (by qPCR) that the expression levels of *acrA, acrB, acrZ, mdtE*, and *mdtF* increased by 1.2–1.7-fold in the mutants D_25_-C_4_-1d. More importantly, the aforementioned mutants completely lost chloramphenicol resistance after the genes *acrA, acrB, acrZ, mdtE*, or *mdtF* were knocked out (Table S14). Therefore, this study showed that the upregulation of the efflux pump-associated genes AcrAB-TolC and *mdtEF* play key roles in helping *E. coli* mutants resist chloramphenicol.

## Discussion

The overuse or misuse of antibiotics was thought to be the main driver inducing the development of antibiotic resistance in pathogenic bacteria.^1^ However, with the findings that antidepressant fluoxetine can promote the resistance of *E. coli* to multiple antibiotics,^33^ ranking potential risk factors that may enable the development of antibiotic resistance in the gut microbiota, which is becoming a worldwide concern, is urgently required. In this study we describe that the combined exposure to duloxetine and chloramphenicol synergistically promote the development of multi-antibiotic resistance in *E. coli*. In a sense, this suggests that the combined exposure to antibiotics and non-antibiotic pharmaceutics in the gut may synergistically accelerate the evolution of ARB, resulting in the exacerbation of the antibiotic resistance crisis in humans.

Generally, gut micribiota may be subjected to short-term high-dose exposure at the level of mg/L due to clinical drug use or long-term low-dose exposure to antibiotics and depressants at the level of μg/L due to residual drugs in the food chain or drinking water.^5,8,16,34^ Maier et al.^35^ deduced the concentrations of drugs in the colon on the basis of the drug excretion patterns from published work and those in the small intestine on the basis of the daily doses of individual drugs consumed. Their findings showed that the media estimated in the small intestine and colon concentrations were higher than 20 μM for both active and inactive compounds, indicating that the simultaneous use of duloxetine and chloramphenicol in clinical trials would lead to a short-term combined exposure to duloxetine and chloramphenicol, at the level of mg/L, to the gut microbiota. In addition, findings from previous studies, which showed that the antibiotic concentration in the meconium reached up to over 150 ng/g (including chloramphenicol),^36^ suggested that the cumulative exposure to low-dose antibiotics was common during pregnancy. A clinical trial found that the antibiotic concentrations in the guts of healthy individuals were 26.51 ± 33.48 ng/g.^7^ Therefore, gut microbiota may have been exposed to both residual non-antibiotic pharmaceuticals and antibiotics, at the level of μg/L, for a long time. Base on these studies we believe that *E. coli* can develop multiple antibiotic resistance after being to duloxetine and chloramphenicol not only in *vitro*, but also in *vivo*.

Previous studies found that the antidepressant sertraline has antimicrobial abilities and exhibits synergistic effects in enhancing the activity of multiple antibiotics through the inhibition of efflux pumps.^37,38^ However, in this study, we found that the enhanced expression of the efflux pumps, including the AcrAB-TolC transport system and the MlaFEDB ABC transporter together via the ROS-induced mutagenesis of *marR*, contribute to synergistic antibiotic resistance. In the multi-drug resistance operon (Mar), which is involved in regulating intrinsic susceptibility to structurally unrelated antibiotics, MarR binds to the *marRAB* promoter and negatively regulates gene expression.^19^ The overexpression of *marA* results in the multiple antibiotic resistance phenotype. Transcription of the *acrAB* operon, whose expression is modulated by global stress signals,^30^ is elevated in strains containing *marR* mutations.^39^ Therefore, the mutation in *marR* here may result in the loss of its DNA-binding ability,^40^ leading to the increased transcription of the *marRAB* and *acrAB* operons via MarA activation, resulting in bacterial resistance to multiple antibiotics. In addition, combined exposure to duloxetine and chloramphenicol resulted in a more robust oxidative stress response in *E. coli* than in the independent study. Above all, the combined group showed a two-fold increase in the ROS content, while the ROS content in the independent exposure group showed no significant difference compared with that in the control group. Considering that oxidative stress in bacteria may cause serious oxidative damage in DNA through nonspecific rapid reactions,^41^ *marR* mutations herein can be induced by ROS produced in the combined exposure group. Previously, Jin et al.^33^ demonstrated that the exposure to the antidepressant fluoxetine induces multiple antibiotic resistance in *E. coli* via the ROS-mediated mutagenesis of DNA-binding transcriptional regulators, which enhances the antibiotic efflux. MlaF is also required for *Acinetobacter baumannii* survival in the presence of sub-inhibitory concentrations of colistin,^42^ and deleting *mlaF* makes *A. baumannii* more susceptible to antibiotics.^43^

The dominant mechanisms underlying the resistance to chloramphenicol in bacteria are enzymatic inactivation by acetylation, clearance via efflux pumps, and ribosome protection.^44–47^ However, in this study, no transcriptional enchancement of chloramphenicol acetyltransferase or ribosome protection were found in the mutants. Interestingly, in all mutants, regardless of whether chloramphenicol resistance was induced by duloxetine, chloramphenicol, or their combination, the mechanism underlying the resistance against chloramphenicol was the same, i.e., the upregulation of the efflux pumps AcrAB-TolC and *mdtEF*. Therefore, herein, the enhanced antibiotic efflux pumps, and not chloramphenicol acetyltransferase or ribosome protection, contributes to the resistance of *E. coli* against chloramphenicol. Multidrug efflux pumps play important roles in the multiple antibiotic resistance of *E. coli*, indicating that it may serve an efficient target to control infections caused by ARB using drug efflux inhibitors.

In conclusion, our findings demonstrated that the combined exposure to duloxetine and chloramphenicol can synergistically promote the development of multi-antibiotic resistance in *E. coli*, indicating that antibiotics and non-antibiotic pharmaceutics can synergistically accelerate the evolution of resistance to multiple antibiotics in bacteria *in vitro*. Moreover, the enhancement of the activities of the AcrAB-TolC transport system and the MlaFEDB ABC transporter via ROS-induced mutagenesis contribute to the synergistic increase in antibiotic resistance in the combined exposure group; further, the improved resistance of the resulting mutants against chloramphenicol involved the improved activities of the efflux pump AcrAB-TolC and mdtEF. These results broaden our understanding of the emergence of antibiotic resistance in the gut and provides a theoretical basis for ARB dissemination in nature.

## Methods

### Bacteria strains, plasmids, and culture media

*E. coli* strain K12 (MG 1655) purchased from the American Type Culture Collection (ATCC 15597) was grown in lysogeny broth (LB) (10 g/L tryptone (Difco), 5 g/L yeast extract (Difco), 10 g/L NaCl) at 37°C. *E. coli* DH5α competent cells purchased from TIANGEN BIOTECH (Beijing, China) were cultured in LB at 37°C. *E. coli* with pRedCas9 plasmid was cultured in LB at 30°C. Table S15 lists the bacteria strains and plasmids used in this study. Table S16 lists the primers used in this study.

### Bacterial exposure to duloxetine and/or chloramphenicol

After *E. coli* strain K12 (MG 1655) was incubated at 37°C for 12 h, 30 µL of fresh cell suspension was added to 2.97 mL LB broth containing duloxetine (2.5 µg/L–100 mg/L) and/or chloramphenicol (6 µg/L–16 mg/L) to reach the final cell density of 10^6^–10^7^ CFU/mL. The *E. coli* cultures were incubated at 37°C for 24 h. Then 30 µL of the cultures were transferred into 2.97 mL fresh LB containing the corresponding concentration of duloxetine and/or chloramphenicol. This subculture process was repeated for up to 50 days. During this process, 100 µL of *E. coli* cultures were spread on M-endo agar containing 16 mg/L chloramphenicol to screen for chloramphenicol-resistant bacteria, and antibiotic-free LB agar, which was used for the total bacterial count. This procedure was performed on Days 1, 3, 5, 10, 20, 30, 40, and 50 and the plates were incubated for 24 h at 37°C. Then, 5–8 chloramphenicol-resistant mutants from each batch were selected at random and stored at −20°C. As controls, *E. coli* cultured in the LB broth without duloxetine or chloramphenicol were also transferred for subculture and then were spread on M-endo agar containing 16 mg/L chloramphenicol to observe spontaneous mutation frequency. All experiments were performed in biological triplicates.

### Calculation of the mutation frequency

The mutation frequency against chloramphenicol was calculated by dividing the number of chloramphenicol-resistant colonies by the total bacterial count using Equation 1.

(1) $$f=\;\frac{N_{0}}{N}$$

Where N_0_ refers to the number of chloramphenicol-resistant bacteria and N refers to total bacteria count.

### Evaluation of the combined effect of duloxetine and chloramphenicol

To evaluate the combined effects of duloxetine and chloramphenicol, the combination index was calculated using the modified Burgi formula (i.e., Jin equation)^48^ here written as Equation 2, which is universally accepted in pharmacology.

(2) $$q=\frac{f_{C+D}}{f_{C}+f_{D}-f_{C}\times f_{D}}$$

where *f*_C_, *f*_D_, and *f*_C+D_, refer to the mutation frequency of *E. coli* independently exposed to chloramphenicol, duloxetine, and their combined exposure, respectively. The expected value q represents the combination index, 0.85 < q < 1.15 would mean addition, q > 1.15 means synergism, and q < 0.85 means antagonism.

### Antibiotic susceptibility testing

The MICs of the resistant mutants *E. coli* against 29 different antibiotics were determined using the Thermo Scientific^TM^ Sensititre^TM^ Susceptibility Testing System (Thermo Fisher Scientific, USA) according to the manufacturer’s instructions. Supplementary Text 1 introduced the detailed procedures.

To test the hereditary stability of the resistant mutant *E. coli*, the resistant mutants were cultured overnight in LB then inoculated into fresh 1% (V/V) LB for continuous overnight culture. After ten days of repeated subculture, the antibiotic susceptibility of the cells was determined as described above.

### Nucleic acid extraction and sequencing

Bacterial DNA was extracted from wild-type *E. coli* K12 or mutants cultured in LB broth for 10 h at 37°C using the FastDNA^TM^ SPIN kit (MP Biomedicals, CA, USA). For mutants isolated from the same conditions, three clones were selected for DNA sequencing. Bacterial RNA from wild-type *E. coli* K12 or mutants cultured in the LB broth containing chloramphenicol and/or duloxetine for 10 h at 37°C were extracted with the EZ-10 Spin Column Total RNA Isolation Kit (BBI Lifesciences, USA). DNA samples (A260/A280, 1.8–2.0) and RNA samples (RIN ≥ 7) were submitted to Novogene Co. (China) and sequenced on a MiSeq instrument (Illumina) with 150 bp double-end sequencing with coverage of >100-fold. Supplementary Text 4 describes the procedures in detail.

### RNA-Seq data processing and global transcriptional analysis

RNA sequencing and differential expression analysis were performed in accordance with previous methods.^49^ Gene expression level was quantified using HTSeq (Version 0.6.1). Differential expression analysis of the groups was performed using the DESeq R package (1.18.0). The resultant *P*-values were adjusted using Benjamini and Hochberg’s approach for controlling the false discovery rate. Genes with significant fold differences (log_2_ > 1 or log_2_ < −1) and adjusted *P*-value <0.05 found by DESeq were assigned as differentially expressed. The data visualization tool Rstudio (Version 1.2.1335) was used for comprehensive comparison and graphical drawing.

### DNA-seq data processing and mutation analysis

Genomic alignment and mutation analysis were performed according to the previous method^50^ with reference to the genome of *E. coli* K12 strain (Genbank accession number, U00096.3). Brimmy Trimmomatic (version 0.36) was used to trim Illumina’s paired-end raw data, leaving only the correctly paired readings for further downstream analysis. To study small variations, these high read depth data sets were aligned to the reference sequence using BWA (Version: 0.7.8)^51^ and SAMTOOLS (Version: 0.1.18), and then the highly divergent loci were further analyzed. The data visualization tools Rstudio (Version 1.2.1335) and Circos (Version 0.64) were used for comprehensive comparison and graphical drawing.

### Quantification of gene expression by RT- qPCR

Relative gene expression in bacteria was quantified by RT-qPCR using One-step TB green RT-PCR kits (Takara, USA). Briefly, 2 μL of RNA samples from bacteria and 0.4 μL of each primer (0.2 μM) were added to a 17.2 μL reaction mixture containing 10 μL 2 × One-step TB Green RT-PCR BufferI III, 0.4 μL PrimeScript RT enzyme Mix II, 0.4 μLTaKaRa Ex TaqHS (5 U/μL), 0.4 μL ROX Reference Dye II (50×), and 6 μL RNase-free water. The reaction conditions were 42°C for 5 min, 95°C for 10 s, followed by 40 cycles of 95°C for 5 s and 60°C for 34 s. The reactions were performed in an ABI Viia7 sequence detection system (Applied Biosystems, USA) in triplicate. The *E. coli* 16s RNA genes were used as a housekeeping gene. The fold difference of the genes was calculated using the comparative ∆CT method (Equation 3).^52^ Table S16 lists the primers used in this study.

(3) $$F=\;2^{-\left[CT_{\left(GOI.sample\right)}-CT_{\left(16s\;RNA.sample\right)}\right]-\left[CT_{\left(GOI.control\right)}-CT_{\left(16s\;RNA.control\right)}\right]}$$

Where F represents the fold difference, CT_(*GOI.sample*)_ and CT_(*16s RNA.sample*)_ represent the CT value of the gene of interest and of the 16s RNA genes in the sample group, respectively, and CT_(*GOI.control*)_ and CT_(*16s RNA.control*)_ represent the CT value of the gene of interest and 16s RNA genes in the control group, respectively.

### Gene knockout by CRISPR/Cas9

Genes *marR, marA, AcrA, acrA, acrB, mlaD, mlaE* were knocked out from wild-type *E. coli* K12 to verify their contribution to antibiotic resistance development during exposure to duloxetine and/or chloramphenicol. Genes *acrA, acrB, mdtE, mdtF, acrZ* were knocked out form *E. coli* mutants selected form drug exposure to verify their resistance mechanisms against chloramphenicol. Supplementary Text 5 describes the process of gene knockout in detail.

### Reactive oxygen species (ROS) and antioxidant measurement

Bacterial ROS were quantified by flow cytometry (BD FACS Calibur, USA) using the DCF-DA/H_2_DCFDA-cellular ROS detection assay kit (Abcam, USA). The activity of superoxide dismutase (SOD), catalase (CAT), glutathione peroxidase (GSH-PX), and total antioxidant capacity (T-AOC) in bacteria were quantified in SpectraMax M5 (Molecular Devices, USA) using the corresponding detection kits from Nanjing Jiancheng Lnc, China. They were all performed following the manufacturer’s instructions; Supplementary Texts 2 and 3 introduce detailed procedures. All measurements were performed in biological triplicate.

## Supplementary Material

Supplemental Material

## Data Availability

DNA and RNA sequencing data are accessible through the Sequence Read Archive (SRA accession number PRJNA698263, PRJNA725495).

## Statistical analysis

Statistical analyses were performed using OriginPro 2018C (OriginLab Corporation, USA), SPSS 20.0 (IBM Corporation, USA), and Rstudio (Version 1.2.1335). Student’s T-test was used to compare the difference in mutation frequency, ROS, antioxidant levels, MICs, and mutant gene levels between treatment groups. For all tests, only data resulting in *P* values < .05 were regarded as statistically significant.
