# Supplementary material for: Combined exposure to non-antibiotic pharmaceutics and antibiotics in the gut synergistically promote the development of multi-drug-resistance in Escherichia coli
Source: Gut Microbes. 2022 Jan 11;14(1):2018901. doi: 10.1080/19490976.2021.2018901 (PMC8757474; doi:10.1080/19490976.2021.2018901)
Supplement: Supplemental Material [file KGMI_A_2018901_SM6769.zip › supplementary/supplemetary material..pdf]

Combined exposure to non-antibiotic pharmaceutics and antibiotics in the gut synergistically promote the development of multi-drug-resistance in *Escherichia coli*

Danyang Shi[a,#], Han Hao[a,#], Zilin Wei[a], Dong Yang[a], Jing Yin[a], Haibei Li[a], Zhengshan Chen[a], Zhongwei Yang[a], Tianjiao Chen[a], Shuqing Zhou[a], Haiyan Wu[a], Junwen Li[a], Min Jin[a,*]

[a] Key Laboratory of Risk Assessment and Control for Environment & Food Safety, Tianjin Institute of Environmental ＆Operational Medicine, No.1, Dali Road, Tianjin 300050, China

[#]Contributed equally to this work.

[*] Correspondence: jinminzh@126.com;

Contents of Supplementary Material

Supplementary Text 1: MIC measurement of *E. coli* against antibiotics.

Supplementary Text 2: ROS detection in the bacteria.

Supplementary Text 3: SOD, CAT, GSH-PX and T-AOC detection in bacteria.

Supplementary Text 4: DNA and RNA sequencing.

Supplementary Text 5: Knock out of the key genes by CRISPR/Cas9.

Figure 1 Global transcriptomic responses of antibiotic-resistant genes (ARG), nucleic acid synthesis and repair-related genes (NSRG), and oxidative stress genes (OSG) in *E. coli* after independent exposure to 25 mg/L duloxetine or 4 mg/L chloramphenicol, or combined exposure.

Table S1 Fold change in the mutation frequencies of chloramphenicol-resistant *E. coli* treated with different dose and time exposures of chloramphenicol relative to the spontaneous mutation frequency of *E. coli*.

Table S2 Fold change in the mutation frequencies of chloramphenicol-resistant *E. coli* treated with different dose and time exposures of duloxetine relative to the spontaneous mutation frequency of *E. coli*.

Table S3 Fold change of the mutation frequencies of chloramphenicol-resistant *E. coli* treated with different dose and time exposures to duloxetine and chloramphenicol, relative to the spontaneous mutation frequency of *E. coli*.

Table S4 MICs against representative antibiotics of *E. coli* mutants isolated from combined or independent exposure to duloxetine and chloramphenicol. (n = 3-5)

Table S5-1 Significance levels of the MICs of *E. coli* mutants isolated from combined or independent exposure to duloxetine and chloramphenicol.

Table S5-2 Significance levels of the MICs of wild *E. coli* K12 and *E. coli* mutants isolated from combined or independent exposure to duloxetine and chloramphenicol.

Table S6 Changes in the expression of genes related to antibiotic resistance in *E. coli* after combined or independent exposure to 25 mg/L duloxetine and 4 mg/L chloramphenicol, relative to *E. coli* without exposure.

Table S7 Significance levels of the gene expression in *E. coli* that treated with 25 mg/L duloxetine or/and 4 mg/L chloramphenicol exposure by (RT-) qPCR.

Table S8 Probability of the increased mutation frequency in *E. coli* with different knockout genes when exposed to duloxetine and/or chloramphenicol (n = 9).

Table S9 Changes in the expression of genes related to oxidative stress in *E. coli* after combined or independent exposure to 25 mg/L duloxetine and 4 mg/L chloramphenicol, relative to *E. coli* without exposure.

Table S10 Fold change of ROS, SOD, CAT, GSH-PX and T-AOC in bacteria exposed to 25 mg/L duloxetine and/or 4 mg/L chloramphenicol, relative to *E. coli* without exposure.

Table S11 Changes in the expression of genes related to DNA repair and DNA replication in *E. coli* after combined or independent exposure to 25 mg/L duloxetine and 4 mg/L chloramphenicol, relative to *E. coli* without exposure.

Table S12 Changes in the expression of genes related to resistance mechanisms against chloramphenicol in *E. coli* mutants after exposure to 4 mg/L chloramphenicol, relative to wild-type *E. coli*.

Table S13 Changes in the expression of genes related to stress defense in *E. coli* mutants after exposure to 4 mg/L chloramphenicol, relative to wild-type *E. coli*.

Table S14 MICs of gene-knockout mutant isolates against chloramphenicol.

Table S15 Strains and plasmids used in this study.

Table S16 Primers used in this study.

Supplementary Text 1: MIC measurement of *E. coli* against antibiotics.
Briefly, 5–8 resistant mutants were selected from fresh primary agar plate, emulsified in sterile water, and adjusted to a 0.5 McFarland Standard bacteria concentration using a Sensititre nephelometer. They were mixed well, and 10 μL of the suspension was transferred into a tube of cation-adjusted Mueller–Hinton broth with TES (N-Tris(hydroxymethyl)methyl-2-aminoethanesulfonic acid) buffer to give an inoculum of 10^5 CFU/mL. Then, 50 μL of the suspension was transferred into Sensititre Susceptibility Testing plates (CHN1GOVF and CNAGOV2F) containing 29 kinds of serially diluted antibiotics. After incubation at 37°C for 24 h, the MICs were measured using the Sensititr™ manual viewer vizion. For each experiment, *E. coli* ATCC 15597 without any drug exposure were used as a negative control.

Supplementary Text 2: ROS detection in the bacteria.
ROS in the bacteria were detected as described below. Briefly, 500 μL of bacteria suspension (10^6 CFU/mL) containing 10 mM DCF-DA was incubated for 45 min at 37°C. DCF-DA loaded cells were then treated with duloxetine (25 mg/L) and/or chloramphenicol (4 mg/L) for 2 h at 30°C. Sterile distilled water was set as a blank control. All samples were scanned by a Flow Cytometer (BD Accuri™, USA) under conditions of excitation at 488 nm and emission at 535 nm. Intracellular levels of ROS were calculated using FlowJo software.

Supplementary Text 3: SOD, CAT, GSH-PX and T-AOC detection in bacteria.
Briefly, 30 μL of overnight-cultured bacteria (10^8–10^9 CFU/mL) was added to 3 mL of fresh LB medium containing duloxetine (25 mg/L) and/or chloramphenicol (4 mg/L) and incubated at 37°C for 10 h. The suspension was centrifuged at 10,000 × *g*, 4°C for 1 min and the cells were added to 1 mL of 0.2 mol/L phosphate-buffered saline (PBS). The suspended solution was sonicated (crushing 4 s, intermittent 5 s, total time 3 min, power 300 W) using an ultrasonic crusher (Sonic, USA) under an ice-water bath and then centrifuged at 5,000 × *g*, 4°C for 3 min. The contents of SOD, CAT, and GSH-Px in the supernatant were quantified using appropriate kits obtained from the Nanjing Jiancheng Bioengineering Institute (Nanjing, China) by following the manufacturers' instructions at the absorbance values of 405 (CAT), 412 (GSH-PX), and 550 nm (SOD), respectively. Bacterial suspensions without any exposure were also observed as controls.

Supplementary Text 4: DNA and RNA sequencing.

To analyze the genetic mutations in bacteria, 10^7–10^8 CFU wild-type *E. coli* K12 in triplicate or three clones of mutants (labeled D_{25}-C_4-1d) were cultured in 10 mL LB broth for 10 h at 37°C with shaking (150 rpm) and the bacterial DNA were extracted using the FastDNA™ SPIN kit (MP Biomedicals, CA, USA) according to the manufacturer's instructions.

To study the synergistic mechanism of duloxetine and chloramphenicol promoting chloramphenicol resistance in *E. coli*, LB broth containing 25 mg/L duloxetine and/or 4 mg/L chloramphenicol was inoculated with 10^6–10^7 CFU/mL freshly cultured wild-type *E. coli* K12, which was performed in triplicate. After incubating for 10 hours at 37°C with shaking (150 rpm), bacterial RNA was extracted from the prepared cells using the EZ-10 Spin Column Total RNA Isolation Kit (BBI Lifesciences, USA) according to the manufacturer's instructions. In parallel, wild-type *E. coli* K12 free of any exposure was used as a background control in triplicate.

To investigate the resistance mechanisms against chloramphenicol for *E. coli* mutants isolated from combined or independent exposure, 10^6–10^7 CFU/mL freshly cultured *E. coli* mutants (labeled D_{100}-1d, C_4-3d, and D_{25}-C_4-1d) and wild-type *E. coli* K12 were inoculated into LB broth containing 4 mg/L chloramphenicol, which was performed in triplicate. After incubating for 10 hours at 37°C with shaking (150 rpm), bacterial RNA was extracted from the prepared cells using the EZ-10 Spin Column Total RNA Isolation Kit (BBI Lifesciences, USA) according to the manufacturer's instructions.

The total DNA and RNA obtained above were subjected to quality control by agarose gel electrophoresis and quantified by Qubit (ThermoFisher Scientific, USA). DNA samples (A260/A280, 1.8–2.0) and RNA samples (RIN ≥ 7) and then submitted to Novogene (Beijing, China) for sequencing. RNA sequencing libraries were generated using NEBNext® Ultra™ Directional RNA Library Prep Kit for Illumina® (NEB, USA) following the manufacturer's recommendations and index codes were added to attribute sequences to each sample. All nucleic acid samples were used for the double-end sequencing of Illumina (PE150, Illumina Inc., San Diego, Calif.).

Supplementary Text 5: Knock out of the key genes by CRISPR/Cas9.
First, gRNA plasmids were constructed. A pair of single-strand DNA sequences were synthesized and annealed to form the dsDNA, which were ligated with linearized pGRB by homologous recombination using the ClonExpress® II One-step Cloning Kit (Vazyme, China). Then, the donor DNA for gene editing

was prepared. The up- and downstream homologous sequences were amplified by PCR, and the protospacer and PAM sequences were synthesized in the primers. The two PCR products were then ligated by overlapping PCR. The gene-editing procedure was performed as follows. The plasmid pREDCas9 was transformed into the *E. coli* K12 competent cells, and single colonies were cultured and identified by PCR. Colonies with correct PCR sequences were cultured overnight in LB medium at 32°C and transferred into 2×YT medium (1.6% peptone, 1% yeast extract, 0.5% NaCl). Then, 0.1 mM IPTG was added when the cells had grown to an OD of 0.1–0.2. The cells were harvested when they reached an OD of 0.4–0.5 to prepare the electroporation-competent cells. In total, 200 ng donor dsDNA and 100 ng gRNA plasmid were added to the electroporation reaction. After electroporation, the cells were added to 1 mL LB and recovered at 32°C for 2 h before planting on LB agar with ampicillin and spectinomycin. The singles were verified by PCR and the positive transformants were confirmed by DNA sequencing. Finally, the gRNA plasmids were cured by incubation in LB containing 0.2% L-arabinose and cultured for 6–8 h. pREDCas9 was cured by culturing overnight at 42°C.

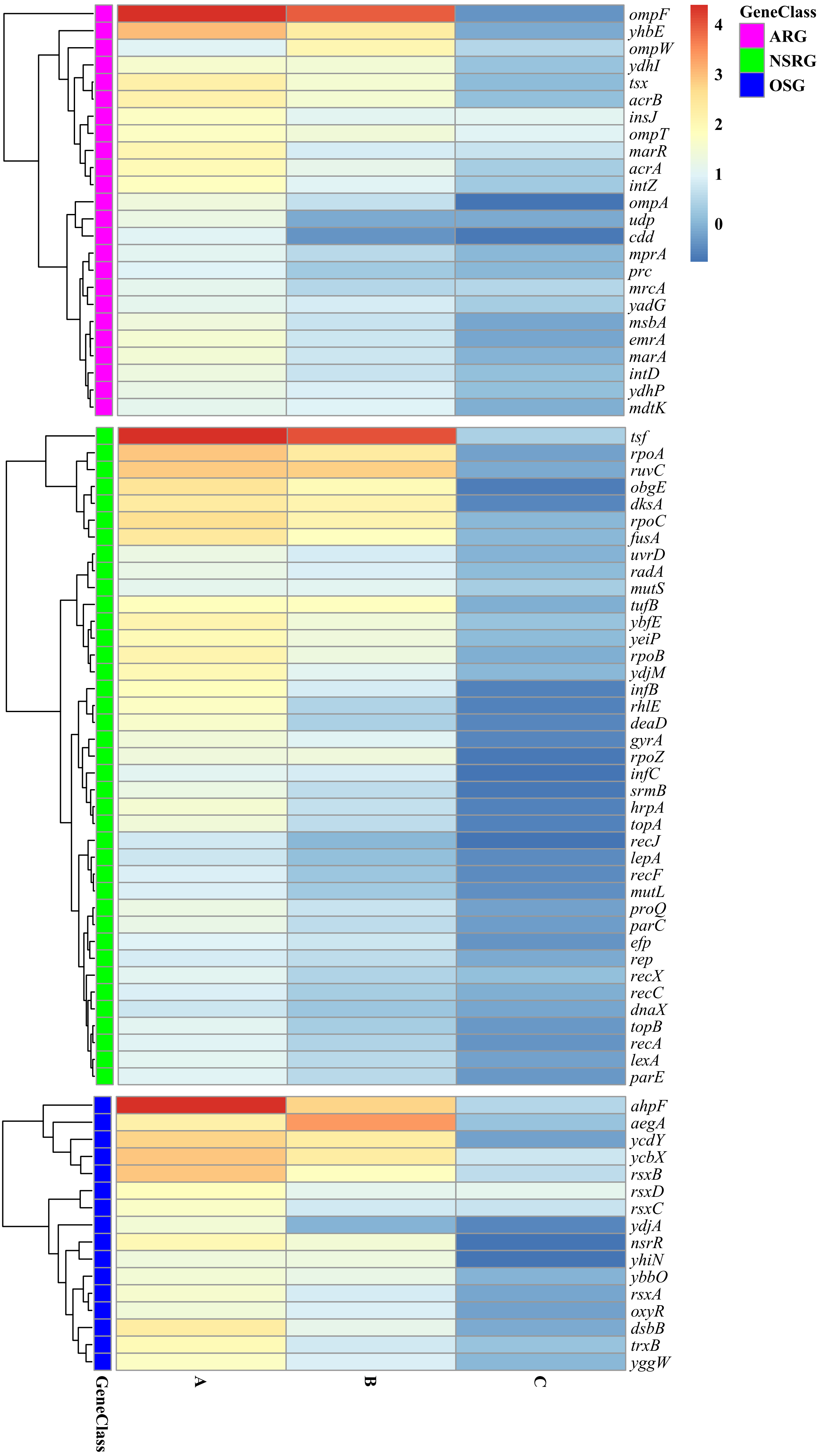

Figure S1. Global transcriptomic responses of antibiotic-resistant genes (ARG), nucleic acid synthesis and repair-related genes (NSRG), and oxidative stress genes (OSG) in *E. coli* after independent exposure to 25 mg/L duloxetine or 4 mg/L chloramphenicol, or combined exposure. Here, 10 mL of LB broth with a mixture of 4 mg/L chloramphenicol and 25 mg/L duloxetine or 25 mg/L duloxetine or 4 mg/L chloramphenicol alone were inoculated with 10^6–10^7 CFU/mL of freshly cultured wild-type *E. coli* K12. Following incubation at 37°C for 10 h with shaking (150 rpm), bacteria were harvested by centrifugation at 8000 × g for 10 min. Then bacterial RNA was extracted for sequencing. The lanes show the fold changes in gene expression between *E. coli* treated with combined (A), 25 mg/L duloxetine (B), or 4 mg/L chloramphenicol (C) exposure and the control (no exposure), respectively.

Table S1 Fold change in the mutation frequencies of chloramphenicol-resistant *E. coli* treated with different dose and time exposures of chloramphenicol relative to the spontaneous mutation frequency of *E. coli*.

Time (Day)	Chloramphenicol concentration (mg/L)					
	16	8	4	2	1	0.5
1	6.5×10^{3}	1.5×10^{3}	2.2×10^{1}	1.0	1.0	1.0
3	4.0×10^{6}	4.1×10^{5}	2.3×10^{4}	1.2×10^{3}	1.0	1.0
5	7.0×107	7.0×10^{7}	5.9×10^{5}	2.9×10^{4}	4.4×10^{2}	1.0

Table S2 Fold change in the mutation frequencies of chloramphenicol-resistant *E. coli* treated with different dose and time exposures of duloxetine relative to the spontaneous mutation frequency of *E. coli*.

Time (Day)	Duloxetine concentration (mg/L)		
	100	50	25
1	3.0×10^{3}	1.0	1.0
3	7.5×10^{4}	1.0	1.0
5	1.3×10^{5}	7.0	1.0

Table S3 Fold change of the mutation frequencies of chloramphenicol-resistant *E. coli* treated with different dose and time exposures to duloxetine and chloramphenicol, relative to the spontaneous mutation frequency of *E. coli*.

Chloramphenicol Concentration (mg/L)	Time (Day)	Duloxetine concentration (mg/L)				
		25	2.5	0.25	0.025	0.0025
4	1	9.1	1.0	1.0	1.0	1.0
	3	1.4×10^{2}	1.4×10^{1}	1.3×10^{1}	1.3×10^{1}	1.0
	5	4.7	1.4	1.2	1.0	1.0
1	1	1.0	1.0	1.0	1.0	1.0
	3	3.8×10^{2}	3.1×10^{2}	2.6	2.4	1.0
	5	3.1	2.5	2.3	1.9	1.0

Table S4 MICs against representative antibiotics of *E. coli* mutants isolated from combined or independent exposure to duloxetine and chloramphenicol. (n = 3-5)

		1st generation, mg/L, mean (SD)						10th generation, mg/L, mean (SD)						CLSI standard, mg/L		
Classification	Antibiotics	K12	D_{25}-C_4-1d	C_4-3d	D_{100}-1d	$D_{0.025}$-$C_{0.06}$-30d	$D_{0.0025}$-$C_{0.006}$-50d	K12	D_{25}-C_4-1d	C_4-3d	D_{100}-1d	$D_{0.025}$-$C_{0.06}$-30d	$D_{0.0025}$-$C_{0.006}$-50d	S	I	R
β-lactams	Ampicillin/ Sulbactam	8	12.8 (4.4)	8 (4)	9.6 (3.6)	8 (0)	9 (2.8)	8	16 (0)	8 (4)	8 (0)	8 (0)	9 (2.8)	≤8/ 4	16/ 8	≥32/ 16
	Cefotaxime	0.25	3.33 (1.2)	0.25 (0)	0.25 (0)	0.92 (1.0)	0.25 (0)	0.25	3.33 (1.2)	0.25 (0)	0.25 (0)	0.25 (0)	0.25 (0)	≤1	2	≥4
	Ceftazidime	0.5	13.3 (4.6)	0.7 (0.3)	0.5 (0)	0.5 (0)	0.7 (0.3)	0.5	13.3 (4.6)	0.7 (0.3)	0.5 (0)	0.5 (0)	0.7 (0.3)	≤4	8	≥16
	Cefazolin	2	32 (0)	1.3 (0.6)	2 (0)	1.3 (0.6)	2 (0)	2	32 (0)	1.33 (0.6)	2 (0)	1.3 (0.6)	2 (0)	≤2	4	≥8
	Cefoxitin	8	64 (0)	21.3 (9.2)	42.7 (18.5)	13.3 (4.6)	21.3 (9.2)	8	64(0)	21.3 (9.2)	42.7 (18.5)	13.3 (4.6)	21.3 (9.2)	≤8	16	≥32
	Ampicillin	2	64 (0)	42.7 (18.5)	26.7 (9.3)	2.7 (1.2)	2 (0)	2	64 (0)	42.7 (18.5)	26.7 (9.3)	2.7 (1.2)	2 (0)	≤8	16	≥32
	Cefotaxime/clavu lanic acid	0.12	0.25 (0)	0.12 (0)	0.17 (0.07)	0.12 (0)	0.15 (0)	0.12	0.25 (0)	0.12 (0)	0.12 (0)	0.12 (0)	0.12 (0)	—	—	—
	Ceftazidime/clav ulanic acid	0.25	0.5 (0)	0.4 (0.1)	0.4 (0.1)	0.4 (0.1)	0.4 (0.1)	0.25	0.5 (0)	0.4 (0.1)	0.4 (0.1)	0.4 (0.1)	0.4 (0.1)	—	—	—
	Cefepime	0.25	0.25 (0)	0.25 (0)	0.25 (0)	0.25 (0)	0.29 (0.09)	0.25	0.25 (0)	0.25 (0)	0.25 (0)	0.25 (0)	0.25 (0)	≤2	—	≥16
	Aztreonam	1	1 (0)	1 (0)	1 (0)	1 (0)	1 (0)	1	1 (0)	1 (0)	1 (0)	1 (0)	1 (0)	≤4	8	≥16
Tetracyclines	Tetracycline	1	32 (0)	32 (0)	13.3 (4.6)	22.7 (16.2)	2 (0)	1	32 (0)	32 (0)	13.3 (4.6)	22.7 (16.2)	2 (0)	≤4	8	≥16
	Doxycycline	2	12.8 (4.4)	6.7 (2.3)	8 (0)	5 (2)	3.8 (0.7)	2	12.8 (4.4)	8 (0)	8 (0)	5 (2)	3.8 (0.7)	≤4	8	≥16
	Minocycline	2	7.2 (1.8)	2.67 (1.2)	6.4 (2.2)	5 (2)	4.5 (2.3)	2	8 (0)	2 (0)	5.2 (1.2)	5 (2)	4.5 (2.3)	≤4	8	≥16
Chloramphenicols	Chloramphenicol	16	64 (0)	53.3 (18.5)	32 (0)	42.7 (18.5)	53.3 (18.5)	16	64 (0)	53.3 (18.5)	32 (0)	42.7 (18.5)	53.3 (18.5)	≤8	16	≥32
	Thiomycin	0.25	0.25 (0)	0.25 (0)	0.25 (0)	0.25 (0)	0.288 (0.09)	0.25	0.25 (0)	0.25 (0)	0.25 (0)	0.25 (0)	0.25 (0)	—	—	—

Quinolones	Nalidixic acid	4	8 (0)	8 (0)	9.6 (3.6)	7 (2)	9 (2.8)	4	8 (0)	8 (0)	8 (0)	7 (2)	9 (2.8)	≤16	—	≥32
	Ciprofloxacin	0.03	0.04 (0.02)	0.04 (0.02)	0.04 (0.02)	0.03 (0)	0.03 (0.01)	0.03	0.04 (0.02)	0.04 (0.02)	0.04 (0.02)	0.03 (0)	0.03 (0.01)	≤0.25	0.5	≥1
	Levofloxacin	0.12	0.12 (0)	0.12 (0)	0.12 (0)	0.12 (0)	0.12 (0)	0.12	0.12 (0)	0.12 (0)	0.12 (0)	0.12 (0)	0.12 (0)	≤0.5	1	≥2
Fluoroquinolone	Gemifloxacin	0.015	0.02 (0.01)	0.02 (0)	0.02 (0.01)	0.02 (0)	0.03 (0.01)	0.015	0.02 (0.01)	0.02 (0)	0.02 (0.01)	0.02 (0)	0.03 (0.01)	≤0.25	0.5	≥1
Macrolides	Azithromycin	4	8 (0)	10.7 (4.6)	8.8 (4.4)	8 (0)	7 (1.9)	4	10.7 (4.6)	8 (0)	8 (0)	8 (0)	7 (1.9)	≤16	—	≥32
Penicillins	Amoxicillin/Clavulanic Acid	8	8 (0)	8 (0)	8 (0)	8 (0)	8 (0)	8	8 (0)	8 (0)	8 (0)	8 (0)	8 (0)	≤8/4	16/8	≥32/16
Dihydropyrimidine	Trimethoprim/Sulfamethoxazole	0.25	0.25 (0)	0.25 (0)	0.25 (0)	0.25 (0)	0.25 (0)	0.25	0.25 (0)	0.25 (0)	0.25 (0)	0.25 (0)	0.25 (0)	≤2/38	—	≥4/76
Aminoglycosides	Kanamycin	1	1.8 (0.5)	1.7 (0.6)	1.6 (0.6)	1 (0)	1.9 (1)	1	1.8 (0.5)	1.7 (0.6)	1.6 (0.6)	1 (0)	1.9 (1)	≤16	32	≥64
Aminoglycosides	Gentamicin	1	1 (0)	1 (0)	1 (0)	1 (0)	1 (0)	1	1 (0)	1 (0)	1 (0)	1 (0)	1 (0)	≤4	8	≥16
	Amikacin	4	4 (0)	4 (0)	4 (0)	4 (0)	4 (0)	4	4 (0)	4 (0)	4 (0)	4 (0)	4 (0)	≤16	32	≥64
Peptides	Polymyxin B	0.5	0.5 (0)	0.5 (0)	0.5 (0)	0.5 (0)	0.5 (0)	0.5	0.5 (0)	0.5 (0)	0.5 (0)	0.5 (0)	0.5 (0)	—	—	—
	Colistin	0.5	0.5 (0)	0.5 (0)	0.5 (0)	0.5 (0)	0.5 (0)	0.5	0.5 (0)	0.5 (0)	0.5 (0)	0.5 (0)	0.5 (0)	≤2	—	≥4
Sulfanilamides	Sulfaisoxazole	32	32 (0)	32 (0)	32 (0)	32 (0)	32 (0)	32	32 (0)	32 (0)	32 (0)	32 (0)	32 (0)	≤256	—	≥512

'—' means not mentioned.

Table S5-1 Significance levels of the MICs of *E. coli* mutants isolated from combined or independent exposure to duloxetine and chloramphenicol.

Classification	Antibiotics	D_{25}-C_4-1d & C_4-3d	D_{25}-C_4-1d & D_{100}-1d	D_{100}-1d & C_4-3d	D_{25}-C_4-1d & $D_{0.025}$-$C_{0.06}$-30d	$D_{0.025}$-$C_{0.06}$-30d & C_4-3d	$D_{0.025}$-$C_{0.06}$-30d & D_{100}-1d	D_{25}-C_4-1d & $D_{0.0025}$-$C_{0.006}$-50d	$D_{0.0025}$-$C_{0.006}$-50d & C_4-3d	$D_{0.0025}$-$C_{0.006}$-50d & D_{100}-1d	$D_{0.0025}$-$C_{0.006}$-50d & $D_{0.025}$-$C_{0.06}$-30d
β-lactams	Ampicillin/Sulbactam	0.038	0.238	0.347	0.038	>0.999	0.347	0.138	0.452	0.776	0.452
	Cefotaxime	0.000	0.000	>0.999	0.007	0.154	0.154	0.000	>0.999	>0.999	0.154
	Ceftazidime	0.000	0.000	0.233	0.000	0.233	>0.999	0.000	0.233	>0.999	>0.999
	Cefazolin	0.000	0.000	0.033	0.000	0.388	0.233	0.000	0.033	>0.999	0.233
	Cefoxitin	0.000	0.033	0.050	0.000	0.122	0.009	0.000	1.000	0.050	0.122
	Ampicillin	0.033	0.000	0.122	0.000	0.001	0.000	0.000	0.001	0.000	0.233
Tetracyclines	Tetracycline	>0.999	0.000	0.000	0.233	0.233	0.250	0.000	0.000	0.001	0.021
	Doxycycline	0.024	0.040	0.233	0.007	0.257	0.010	0.002	0.027	0.000	0.224
	Minocycline	0.001	0.545	0.010	0.104	0.054	0.322	0.074	0.154	0.221	0.725
Chloramphenicols	Chloramphenicol	0.233	0.000	0.033	0.033	0.388	0.233	0.233	1.000	0.033	0.388
Quinolones	Nalidixic acid	>0.999	0.347	0.347	0.296	0.296	0.194	0.452	0.452	0.776	0.233
Macrolides	Azithromycin	0.233	0.694	0.530	>0.999	0.438	0.809	0.262	0.138	0.422	0.717

Table S5-2 Significance levels of the MICs of wild *E. coli* K12 and *E. coli* mutants isolated from combined or independent exposure to duloxetine and chloramphenicol.

Classification	Antibiotics	D_{25}-C_4-1d & K12	C_4-3d & K12	D_{100}-1d & K12	$D_{0.025}$-$C_{0.06}$-30d & K12	$D_{0.0025}$-$C_{0.006}$-50d & K12
β-lactams	Ampicillin/Sulbactam	0.038	>0.999	0.347	>0.999	0.452
	Cefotaxime	0.000	>0.999	>0.999	0.154	>0.999
	Ceftazidime	0.000	0.233	>0.999	>0.999	>0.999
	Cefazolin	0.000	0.033	>0.999	0.233	>0.999
	Cefoxitin	0.000	0.012	0.003	0.033	0.012
	Ampicillin	0.000	0.001	0.000	0.233	>0.999
Tetracyclines	Tetracycline	0.000	0.000	0.000	0.017	0.000
	Doxycycline	0.001	0.002	0.000	0.010	0.001
	Minocycline	0.000	0.233	0.002	0.010	0.043
Chloramphenicols	Chloramphenicol	0.000	0.001	0.000	0.003	0.001
Quinolones	Nalidixic acid	0.000	0.000	0.008	0.010	0.004
Macrolides	Azithromycin	0.000	0.012	0.040	0.153	0.007

Table S6 Changes in the expression of genes related to antibiotic resistance in *E. coli* after combined or independent exposure to 25 mg/L duloxetine and 4 mg/L chloramphenicol, relative to *E. coli* without exposure.

Gene ID	Genes	Fold change			Function
		D_{25}-C_4	C_4	D_{25}	
b0565	*ompT*	3.2*	2.6*	1.0	outer membrane protease VII; outer membrane protein 3b
b0929	*ompF*	21.1*	16.0*	0.8	outer membrane porin 1a
b0957	*ompA*	2.6*	1.6	0.6	outer membrane protein A
b0177	*bamA*	2.3*	1.4	0.9	OM biogenesis outer membrane pore-forming assembly factor
b2477	*bamC*	3.2*	2.5*	0.9	BamABCDE complex OM biogenesis lipoprotein
b0462	*acrB*	4.3*	2.8*	1.1	multidrug efflux system protein
b0463	*acrA*	3.7*	2.3	1.2	multidrug efflux system protein
b1530	*marR*	4.0*	1.7	1.6	transcriptional repressor of multiple antibiotic resistance
b1531	*marA*	2.8*	1.6	1.0	multiple antibiotic resistance transcriptional regulator
b1663	*mdtK*	2.1*	1.9	0.9	multidrug efflux system transporter
b1643	*ydhI*	3.0*	2.8	1.1	DUF1656 family putative inner membrane efflux pump associated protein
b2684	*mprA*	2.1*	1.4	1.0	multidrug efflux
b2685	*emrA*	2.8*	1.6	0.9	multidrug efflux system
b0127	*yadG*	2.1*	1.7	1.3	putative ABC transporter ATPase
b0914	*msbA*	2.6*	1.6	0.9	lipid ABC transporter permease/ATPase
b3193	*mlaD*	3.7*	2.1	1.2	membrane-anchored ABC family periplasmic binding protein
b3194	*mlaE*	3.5*	2.1	1.4	ABC transporter maintaining OM lipid asymmetry
b3195	*mlaF*	4.6*	2.5*	1.4	ABC transporter maintaining OM lipid asymmetry
b1657	*ydhP*	2.3*	1.9	1.1	putative MFS transporter
b0411	*tsx*	4.6*	2.8*	1.0	nucleoside channel
b3184	*yhbE*	8.0*	4.9*	0.9	EamA family inner membrane putative transporter
b2442	*intZ*	3.5*	2.1	1.2	CPZ-55 prophage; putative phage integrase
b0537	*intD*	2.5*	1.6	1.1	DLP12 prophage; putative phage integrase
b3557	*insJ*	3.2*	2.1	2.1	IS150 transposase A
b3396	*mrcA*	2.1*	1.4	1.4	penicillin-binding protein 1a
b1830	*prc*	2.0*	1.2	1.1	carboxy-terminal protease for penicillin-binding protein 3

* means $p < 0.05$ compared with the control group. For the rest, $p > 0.05$ compared with the control group.

Table S7 Significance levels of the gene expression in *E. coli* that treated with 25 mg/L duloxetine or/and 4 mg/L chloramphenicol exposure by (RT-) qPCR.

Genes	Exposure group		
	D_{25}-C_4 & C_4	D_{25}-C_4 & D_{25}	D_{25} & C_4
acrA	<0.001	<0.001	0.003
acrB	<0.001	<0.001	0.101
bamA	0.426	0.700	0.275
bamC	0.099	0.005	0.412
emrA	0.266	0.447	0.088
mlaD	<0.001	0.272	<0.001
mlaE	0.05	0.088	0.05
mlaF	<0.001	0.001	<0.001
marA	<0.001	<0.001	<0.001
marR	<0.001	<0.001	<0.001
mdtK	0.016	<0.001	0.114
msbA	0.009	0.002	0.918
tsx	0.017	0.051	0.491
yadG	0.187	0.206	0.828
ydhP	0.074	<0.001	0.124

Table S8 Probability of the increased mutation frequency in *E. coli* with different knockout genes when exposed to duloxetine and/or chloramphenicol (n = 9).

Gene knockout *E. coli*	Probability of increased mutation frequency (%)		
	25 mg/L duloxetine+4 mg/L chloramphenicol (D_{25}-C_4)	4 mg/L chloramphenicol (C_4)	100 mg/L duloxetine (D_{100})
K12Δ*marA*	0	0	0
K12Δ*acrA*	0	0	33.3
K12Δ*acrB*	16.7	0	33.3
K12Δ*mlaD*	33.3	33.3	16.7
K12Δ*mlaE*	11.1	11.1	11.1
K12Δ*mlaF*	11.1	11.1	11.1
Wild K12	100	100	100

Δ means the gene has been knocked out.

Table S9 Changes in the expression of genes related to oxidative stress in *E. coli* after combined or independent exposure to 25 mg/L duloxetine and 4 mg/L chloramphenicol, relative to *E. coli* without exposure.

Gene ID	Gene	Fold change			Function
		D_{25}-C_4	C_4	D_{25}	
b1627	*rsxA*	2.3*	1.6	1.1	SoxR iron-sulfur cluster reduction factor component
b1628	*rsxB*	4.3*	2.5	1.5	SoxR iron-sulfur cluster reduction factor component
b1629	*rsxC*	2.5*	1.6	1.5	SoxR iron-sulfur cluster reduction factor component
b1630	*rsxD*	2.6*	1.9	1.9	SoxR iron-sulfur cluster reduction factor component
b4063	*soxR*	0.2*	0.2*	0.8	redox-sensitive transcriptional activator of soxS
b0888	*trxB*	2.8*	1.6	1.2	thioredoxin reductase
b0849	*grxA*	27.9*	10.6	3.3	Glutaredoxin; redox coenzyme for ribonucleotide reductase
b0683	*fur*	2.3*	1.3	0.9	ferric iron uptake regulon transcriptional repressor
b0606	*ahpF*	8.6*	4.0*	1.4	alkyl hydroperoxide reductase
b3961	*oxyR*	2.1*	1.7	1.0	oxidative and nitrosative stress transcriptional regulator

* means $p < 0.05$ compared with the control group. For the rest, $p > 0.05$ compared with the control group.

Table S10 Fold change of ROS, SOD, CAT, GSH-PX and T-AOC in bacteria exposed to 25 mg/L duloxetine and/or 4 mg/L chloramphenicol, relative to *E. coli* without exposure.

Exposure group	SOD	ROS	T-AOC	CAT	GSX-PX
D_{25}-C_4	2.8	2	7.7	1.1	29.6
C_4	1.6	1.2	3.4	1	17.8
D_{25}	1	1.5	1.3	1	2.3

Table S11 Changes in the expression of genes related to DNA repair and DNA replication in *E. coli* after combined or independent exposure to 25 mg/L duloxetine and 4 mg/L chloramphenicol, relative to *E. coli* without exposure.

Gene ID	Genes	Fold change			Function
		D_{25}-C_4	C_4	D_{25}	
b4170	*mutL*	2.3*	1.5	0.9	methyl-directed mismatch repair protein
b2733	*mutS*	2.6*	2.5	1.5	methyl-directed mismatch repair protein
b4043	*lexA*	2.6*	1.7	1.1	transcriptional repressor of SOS regulon
b2698	*recX*	2.6*	1.6	1.3	regulatory protein for RecA
b2699	*recA*	2.5*	1.6	0.9	DNA recombination and repair protein; LexA autocleavage cofactor
b2822	*recC*	2.3*	1.6	1.2	exonuclease V (RecBCD complex)
b2892	*recJ*	2.1*	1.2	0.8	ssDNA exonuclease; 5' --> 3'-specific
b3700	*recF*	2.3*	1.4	0.9	gap repair protein
b1084	*rne*	4.9*	3.3*	0.9	Endoribonuclease; RNA degradosome binding protein
b2509	*xseA*	2.6*	1.6	1.0	exonuclease VII
b3813	*uvrD*	2.8*	2.3	1.2	DNA-dependent ATPase I and helicase II
b3183	*obgE*	6.5*	4.6*	0.8	GTPase involved in cell partioning and DNA repair
b0685	*ybfE*	5.3*	3.3*	1.4	LexA-regulated protein
b1728	*ydjM*	4.6*	2.6*	1.2	inner membrane protein regulated by LexA
b4389	*radA*	2.8*	2.3	1.3	DNA repair protein
b1749	*xthA*	2.8*	2.1	1.1	exonuclease III
b4052	*dnaB*	3.3*	2.1	1.1	replicative DNA helicase
b3702	*dnaA*	2.6*	2.1	0.9	chromosomal replication initiator protein DnaA
b3701	*dnaN*	2.6*	2.0	0.8	DNA polymerase III
b0470	*dnaX*	2.1*	1.5	1.1	DNA polymerase III/DNA elongation factor III
b4372	*holD*	3.0*	2.1	0.9	DNA polymerase III
b1099	*holB*	2.3*	1.7	0.9	DNA polymerase III
b2411	*ligA*	6.1*	4.3*	1.4	DNA ligase; NAD (+)-dependent

* means $p < 0.05$ compared with the control group. For the rest, $p > 0.05$ compared with the control group.

Table S12 Changes in the expression of genes related to resistance mechanisms against chloramphenicol in *E. coli* mutants after exposure to 4 mg/L chloramphenicol, relative to wild-type *E. coli*.

Gene ID	Gene	Fold change			Function
		D_{25}-C_4-1d[a]	C_4-3d[b]	D_{100}-1d[c]	
b0792	*ybhR*	4.0	4.0	4.9	putative ABC transporter permease
b0793	*ybhS*	4.6	4.9	6.1	putative ABC transporter permease
b0794	*ybhF*	4.6	4.0	4.9	putative ABC transporter ATPase
b0795	*ybhG*	5.7	5.7	7.0	putative membrane fusion protein (MFP) component of efflux pump
b0127	*yadG*	2.0	1.2	1.5	putative ABC transporter ATPase
b0128	*yadH*	3.0	2.1	2.8	putative ABC transporter permease
b1053	*mdtG*	4.3	4.6	4.9	putative drug efflux system protein
b1065	*mdtH*	2.8	2.5	3.0	multidrug resistance efflux transporter conferring overexpression resistance to norfloxacin and enoxacin
b1599	*mdtI*	9.2	5.3	9.2	multidrug efflux system transporter
b1600	*mdtJ*	11.3	5.7	8.0	multidrug efflux system transporter
b3513	*mdtE*	8.6	14.9	21.1	anaerobic multidrug efflux transporter; ArcA-regulated
b3514	*mdtF*	2.0	2.5	3.3	anaerobic multidrug efflux transporter; ArcA-regulated
b4337	*mdtM*	12.1	11.3	18.4	multidrug efflux system protein
b3009	*yghB*	2.6	2.5	3.5	putative multidrug efflux transporter
b3095	*yqjA*	2.3	1.6	2.3	putative multidrug efflux transporter
b3754	*hsrA*	7.0	6.1	9.2	putative multidrug or homocysteine efflux system
b0878	*macA*	2.0	1.3	1.6	macrolide transporter membrane fusion protein (MFP) component
b0879	*macB*	8.0	8.6	10.6	macrolide ABC transporter peremase/ATPase
b3487	*yhiI*	4.6	5.3	6.5	putative membrane fusion protein (MFP) of efflux pump
b3597	*yibH*	2.1	3.7	4.9	putative membrane fusion protein (MFP) component of efflux pump
b0898	*ycaD*	2.6	2.6	3.7	putative MFS transporter
b1530	*marR*	18.4	10.6	17.2	transcriptional repressor of multiple antibiotic resistance
b1531	*marA*	13.0	7.5	17.2	multiple antibiotic resistance transcriptional regulator
b1532	*marB*	10.6	7.0	13.9	periplasmic mar operon regulator

b0762	*acrZ*	7.5	4.3	4.9	AcrAB-TolC efflux pump accessory protein%2C membrane-associated
b3357	*crp*	2.1	1.6	1.9	cAMP-activated global transcription factor%2C mediator of catabolite repression
b4396	*rob*	3.0	2.6	4.3	right oriC-binding transcriptional activator%2C AraC family
b3067	*rpoD*	3.0	5.3	4.6	RNA polymerase%2C sigma 70 (sigma D) factor
b0814	*ompX*	2.0	1.4	1.9	outer membrane protein X
b2215	*ompC*	2.1	2.1	2.3	outer membrane porin protein C
b0111	*ampE*	2.5	2.1	2.1	ampicillin resistance inner membrane protein; putative signaling protein in beta-lactamase regulation

p (a VS b) > 0.05 ; p (a VS c) > 0.05.

Table S13 Changes in the expression of genes related to stress defense in *E. coli* mutants after exposure to 4 mg/L chloramphenicol, relative to wild-type *E. coli*.

Gene ID	Gene	Fold Change			Function
		D_{25}-C_4-1d[a]	C_4-3d[b]	D_{100}-1d[c]	
b4063	*soxR*	6.8	9.9	6.8	redox-sensitive transcriptional activator of soxS
b4189	*bsmA*	2.1	2.7	4.7	bioflm peroxide resistance protein
b3238	*yhcN*	26.9	24.3	32.9	cadmium and peroxide resistance protein
b0917	*ycaR*	2.8	1.9	1.3	peroxide and acid resistance protein
b0491	*fetB*	2.4	2.0	2.4	peroxide resistance protein
b0490	*fetA*	2.2	2.1	2.9	peroxide resistance protein
b2582	*trxC*	1.9	2.0	3.0	thioredoxin 2
b2922	*yggE*	4.3	3.9	5.3	oxidative stress defense protein
b3153	*yhbO*	2.6	4.2	6.2	stress-resistance protein
b2670	*alaE*	17.8	16.2	13.7	stress-responsive
b0607	*uspG*	15.1	12.2	18.4	universal stress protein UP12
b1376	*uspF*	9.5	15.3	22.4	stress-induced protein
b3923	*uspD*	8.8	10.6	16.5	stress-induced protein
b1333	*uspE*	7.8	6.5	9.5	stress-induced protein
b3495	*uspA*	7.1	6.5	6.6	universal stress global response regulator
b0812	*dps*	6.9	5.1	8.2	stress-inducible DNA-binding protein
b4484	*cpxP*	19.7	8.4	11.8	inhibitor of the cpx response; periplasmic adaptor protein
b1283	*osmB*	6.2	12.8	21.5	osmotically and stress inducible lipoprotein
b3494	*uspB*	6.1	9.3	18.7	universal stress (ethanol tolerance) protein B
b1895	*uspC*	5.3	4.7	6.4	universal stress protein

p (a VS b) > 0.05 ; p (a VS c) > 0.05.

Table S14 MICs of gene-knockout mutant isolates against chloramphenicol.

Gene-knockout mutant isolates	MIC against Chl (mg/L)
D_{25}-C_4-1dΔ*acrA*	4
D_{25}-C_4-1dΔ*acrB*	4
D_{25}-C_4-1dΔ*acrZ*	4
D_{25}-C_4-1dΔ*mdtE*	4
D_{25}-C_4-1dΔ*mdtF*	4
C_4-3dΔ*acrA*	4
C_4-3dΔ*acrB*	4
C_4-3dΔ*acrZ*	4
C_4-3dΔ*mdtE*	4
C_4-3dΔ*mdtF*	4
D_{100}-1dΔ*acrA*	4
D_{100}-1dΔ*acrB*	4
D_{100}-1dΔ*acrZ*	4
D_{100}-1dΔ*mdtE*	4
D_{100}-1dΔ*mdtF*	4
Wild *E.coli* K12	16

Δ means the gene has been knocked out.

Table S15 Strains and plasmids used in this study.

Strains	Characteristics	Source
E. coli K12	Wild type	ATCC
E. coli DH5α	F^- φ80d*lacZ*ΔM15 Δ(lac*ZYA*-*argF*)U169 *recA1 endA1* *hsdR17* (r_k^-,m_k^+)*deoR supE44* λ^- *thi-1 gyrA*96 *relA1*	TIANGEN BIOTECH
E. coli marR	*MarR* DNA fragment knockout *E. coli* K12	This study
E. coli marA	*MarA* DNA fragment knockout *E. coli* K12	This study
E. coli acrA	*AcrA* DNA fragment knockout *E. coli* K12	This study
E. coli acrB	*AcrB* DNA fragment knockout *E. coli* K12	This study
E. coli acrZ	*AcrZ* DNA fragment knockout *E. coli* K12	This study
E. coli mlaD	*mlaD* DNA fragment knockout *E. coli* K12	This study
E. coli mlaE	*mlaE* DNA fragment knockout *E. coli* K12	This study
E. coli mlaF	*mlaF* DNA fragment knockout *E. coli* K12	This study
E. coli mdtE	*mdtE* DNA fragment knockout *E. coli* K12	This study
E. coli mdtF	*mdtF* DNA fragment knockout *E. coli* K12	This study
Plasmids		
pREDCas9	Spe^r, Cas9 and λ-Red recombinase expression vector	Prof. Tao Chen of Tianjin University
pGRB	Amp^r, gRNA expression vector	Prof. Tao Chen of Tianjin University
pGRB-*marR*	pGRB with *MarR* protospacer sequence	This study
pGRB-*marA*	pGRB with *MarA* protospacer sequence	This study
pGRB-*acrA*	pGRB with *AcrA* protospacer sequence	This study
pGRB-*acrB*	pGRB with *AcrB* protospacer sequence	This study
pGRB-*acrZ*	pGRB with *AcrZ* protospacer sequence	This study
pGRB-*mlaD*	pGRB with *mlaD* protospacer sequence	This study
pGRB-*mlaE*	pGRB with *mlaE* protospacer sequence	This study
pGRB-*mlaF*	pGRB with *mlaF* protospacer sequence	This study
pGRB-*mdtE*	pGRB with *mdtE* protospacer sequence	This study
pGRB-*mdtF*	pGRB with *mdtF* protospacer sequence	This study

Table S16 Primers used in this study.

Primers	Sequence (5′-3′)	Source
acrB-F	CGTAAGTCGATGGGGCAGA	This study
acrB-R	CACCAGTAGAACCGCCAAAGA	This study
acrA-F	ATCAGGCTCTGGCTGATGC	This study
acrA-R	TCGGAGAGGTGACTTTGGTGT	This study
marR-F	CCTGCTTAACGAGTATCTGTCTCC	This study
marR-R	ACCTTTCCACCCAGCCTTTAC	This study
marA-F	ATCCGCAGCCGTAAGATGA	This study
marA-R	GGTTCGGGTCAGAGTTTGTTG	This study
ydhP-F	GCAACGTACTTTCCGCCATC	This study
ydhP-R	CACGACTGAACCCAAACCAA	This study
mdtK-F	CTGCGGTTATGAAACGACTGA	This study
mdtK-R	GGAGACACTAACAGAGCCACGA	This study
yadG-F	TAATGATTGCCCGTGCGTTA	This study
yadG-R	TGGTGGTGCCTTTGTCGTT	This study
msbA-F	GGTCAGGAAGTGGAAACGAAAC	This study
msbA-R	TCGCCGCATACAGAACAAAC	This study
bamC-F	CAGCGTTACAGCACGGAGA	This study
bamC-R	TAGTGGTGGAGGCACGATTTT	This study
bamA-F	CGCTGGGGTTATGGTGATG	This study
bamA-R	GCCTGATGCGGGAAGTAAA	This study
mlaD-F	GTCAGTATTGGTGGCGTTGTTG	This study
mlaD-R	GCGAACTGGTATCTGGAATGTG	This study
mlaE-F	TCTGACCACTTATAGTGCGGAAAC	This study
mlaE-R	GAAATAACCCCAGCCCAGAAA	This study
mlaF-F	TGGTGAGATCCTTTTCGATGGT	This study
mlaF-R	GCGGGAAGTTGGGTATGTTC	This study
tsx-F	CGTAATAAAGATGGTCGCCAGAG	This study
tsx-R	CGTCCCACTCGTTTTCGTT	This study
emrA-F	GGATATTTACGGCGATGATGTG	This study
emrA-R	TTCGATACGCACAGGCAGA	This study
rpoD-F	CGTGCGAAGAAAGAGATGGTT	This study
rpoD-R	CAGACCGATGTTGCCTTCCT	This study
ybhR-F	CGATTCTGATTTTACCCGTGCT	This study
ybhR-R	CGAATGCTCGCCGTTATCTT	This study
ybhS-F	TGGCGATGTTGCTGTGTATG	This study
ybhS-R	GTTGCGGGTAATCGTGGAA	This study
ybhF-F	TCAGGGGAAATCGGTACGTCT	This study
ybhF-R	CCGCCCAGCAAATCAATAAA	This study
ybhG-F	GGTGGACGAAGGTGATGCT	This study
ybhG-R	GAGCGATTTCTTCATTGCGATAC	This study
yadH-F	GTATTTGGGCGGGGTCTTTT	This study
yadH-R	TTGATACCGAGGAAGCCGTAG	This study
mdtE-F	GTTAGCCGTCAGGATTACGACAC	This study
mdtE-R	CAGATTGATCGTCGCCTGTT	This study
mdtF-F	TCAGGAAGCGTTATCCTCAAATC	This study
mdtF-R	AACGGAACAACCAACATCACC	This study
mdtI-F	CGTGCTGGAAATCGTTGCT	This study
mdtI-R	TGGCGGCAATACCAAACC	This study
mdtM-F	CGTGCCGTACCCATTCAAC	This study

mdtM-R	CAAACCAATCCCGAAAGCA	This study
crp-F	GCTGAATCTGGCAAAACAACC	This study
crp-R	CACGAGAACAGCCGACAATC	This study
cpxP-F	TCCTGTTAATGTTAGCGAACTGGA	This study
cpxP-R	TACATTTGGTTGCGGACTTTTG	This study
rob-F	GTGTTACCCAGAGCTACTCCTGTTC	This study
rob-R	GAATACCTCTTGCTCGTCGTCTTT	This study
soxR-F	GGCATACGTTAAGTGCGAAAGAG	This study
soxR-R	CACAACCAATACATCCGTCCA	This study
acrZ-F	GGTCTGATTTACGGTCTTGGTGA	This study
acrZ-R	TATCGGCAGGAAAAGCAGGA	This study
pGRB-*marR*-U	AGTCCTAGGTATAATACTAGTATTGGGTCGCTTAATC CATAGTTTTAGAGCTAGAA	This study
pGRB-*marR*-D	TTCTAGCTCTAAAACTATGGATTAAGCGACCCAATAC TAGTATTATACCTAGGACT	This study
marR-test-U	CTCCGGTGAATCAATCGCTT	This study
marR-test-D	CAACCACGACATTGGCACAA	This study
pGRB-*marA*-U	AGTCCTAGGTATAATACTAGTCCATTCATAGCATTTTG GACGTTTTAGAGCTAGAA	This study
pGRB-*marA*-D	TTCTAGCTCTAAAACGTCCAAAATGCTATGAATGGA CTAGTATTATACCTAGGACT	This study
marA-test-U	TGGGCAATATTATCCCCTGCAA	This study
marA-test-D	TATGGGATCTGGGGGACGTT	This study
pGRB-*acrA*-U	AGTCCTAGGTATAATACTAGTGAAGTTCGTCCTCAA GTTAGGTTTTAGAGCTAGAA	This study
pGRB-*acrA*-D	TTCTAGCTCTAAAACCTAACTTGAGGACGAACTTCA CTAGTATTATACCTAGGACT	This study
acrA-test-U	GCACAACAGCCATTTCTCCG	This study
acrA-test-D	TAACGCCGACAACCATCAGG	This study
pGRB-*acrB*-U	AGTCCTAGGTATAATACTAGTCTGACCTCTACTGAAG AGTTCTTTTAGAGCTAGAA	This study
pGRB-*acrB*-D	TTCTAGCTCTAAAAGAACTCTTCAGTAGAGGTCAGA CTAGTATTATACCTAGGACT	This study
acrB-test-U	ATTGGCGATAAGTGGCTGGT	This study
acrB-test-D	AACAGATAGGCCATGCCGAC	This study
pGRB-*acrZ*-U	AGTCCTAGGTATAATACTAGTGTCGTGATGGCCATCA TCCTGTTTTAGAGCTAGAA	This study
pGRB-*acrZ*-D	TTCTAGCTCTAAAACAGGATGATGGCCATCACGACA CTAGTATTATACCTAGGACT	This study
acrZ-test-U	GAAAAACGTGAGATCGCGGC	This study
acrZ-test-D	GTGTCTGACGCGTAGCTGTA	This study
pGRB-*mlaD*-U	AGTCCTAGGTATAATACTAGTATGGTGCTGGAAGATC TCATGTTTTAGAGCTAGAA	This study
pGRB-*mlaD*-D	TTCTAGCTCTAAAACATGAGATCTTCCAGCACCATAC TAGTATTATACCTAGGACT	This study
mlaD-test-U	ATCGCTACTGCGTGAACTGG	This study
mlaD-test-D	ACGGCCATTCGGGTCAATAA	This study
pGRB-*mlaE*-U	AGTCCTAGGTATAATACTAGTATGTTATTCAATGCGCT GGTGTTTTAGAGCTAGAA	This study
pGRB-*mlaE*-D	TTCTAGCTCTAAAACACCAGCGCATTGAATAACATA CTAGTATTATACCTAGGACT	This study
mlaE-test-U	CCTATCCACTGCGCGAACAT	This study
mlaE-test-D	AGATCTTCCAGCACCATCGC	This study
pGRB-*mlaF*-U	AGTCCTAGGTATAATACTAGTTTCTGAACTTTCCGGT GGGAGTTTTAGAGCTAGAA	This study
pGRB-*mlaF*-D	TTCTAGCTCTAAAACTCCCACCGGAAAGTTCAGAAA CTAGTATTATACCTAGGACT	This study
mlaF-test-U	CGGTAAAAGGACGAACCAGC	This study

mlaF-test-D	GGGAGAAATAACCCGACGCA	This study
pGRB-*mdtE*-U	AGTCCTAGGTATAATACTAGTGAGTTTAGCTCGGCCT GTAAGTTTTAGAGCTAGAA	This study
pGRB-*mdtE*-D	TTCTAGCTCTAAAACTTACAGGCCGAGCTAAACTCA CTAGTATTATACCTAGGACT	This study
mdtE-test-U	ATCACGTCGACGCTCAATATTTC	This study
mdtE-test-D	TAAACGCCGCTACCATCAGG	This study
pGRB-mdtF-U	AGTCCTAGGTATAATACTAGTTCAGCGTGATGGAGA ACTGGGTTTTAGAGCTAGAA	This study
pGRB-*mdtF*-D	TTCTAGCTCTAAAACCCAGTTCTCCATCACGCTGAA CTAGTATTATACCTAGGACT	This study
mdtF-test-U	AACAGGTTCAGGGCAGTACG	This study
mdtF-test-D	ACTATTGCGGAAAAATGTGGCT	This study
pGRB-liner-U	GTTTTAGAGCTAGAAATAGCAAGTTAA	Prof. Tao Chen of Tianjin University
pGRB-liner-D	ATTATACCTAGGACTGAGC	Prof. Tao Chen of Tianjin University
pGRB-U	GTCTCATGAGCGGATACATATTTG	Prof. Tao Chen of Tianjin University
pGRB-D	ATGAGAAAGCGCCACGCT	Prof. Tao Chen of Tianjin University
pRedcas9-U	CAGTGGCGGTTTTCATG	Prof. Tao Chen of Tianjin University
pRedcas9-D	CCTTGGTGATCTCGCCTTTC	Prof. Tao Chen of Tianjin University
